# Polymeric nanocarriers in drug delivery, bioimaging, and biosensing: advances and perspectives on nanomicelles, nanogels, and dendrimers

**DOI:** 10.1039/d5ra05399d

**Published:** 2025-10-23

**Authors:** Mehdi Rajabi, Hossein Feyzbakhsh

**Affiliations:** a Guilan University Guilan Iran mehdi.raajabi72@gmail.com; b Università di Bologna Bologna Italy hossein.feyzbakhsh@studio.unibo.it; c Payame Noor University Rasht Iran

## Abstract

Polymeric nanocarriers such as nanomicelles, nanogels, and dendrimers are critical nanomedicine tools, providing novel drug delivery, bioimaging, and biosensing solutions. The design, synthesis, and potential of polymeric nanocarriers to solve long-term biomedical issues are addressed in this review. Nanomicelles, which are self-assembled from amphiphilic copolymers, increase drug solubility such as paclitaxel and allow stimuli-responsive site-specific release controlled by pH or light. Nanogels comprising water-holding polymer networks deliver and protect a broad variety of agents, ranging from anticancer drugs to insulin, across barriers, such as the blood–brain barrier (BBB). Tree-branched dendrimers enhance drug delivery and contrast imaging through precise molecular conjugation. For bioimaging, these carriers increase the resolution of magnetic resonance imaging (MRI), computed tomography (CT), and near-infrared (NIR) fluorescence imaging, while for biosensing, they enable sensitive detection of glucose and other analytes. Despite their potential, scalability, and clinical translation, challenges exist. This review charts their groundbreaking impact through case studies and paving the way for personalized medicine.

## Abstract 2

This review uniquely integrates polymeric nanocarriers nanomicelles, nanogels, and dendrimers, comparing their design and applications in drug delivery, bioimaging, and biosensing.

## Graphical abstract

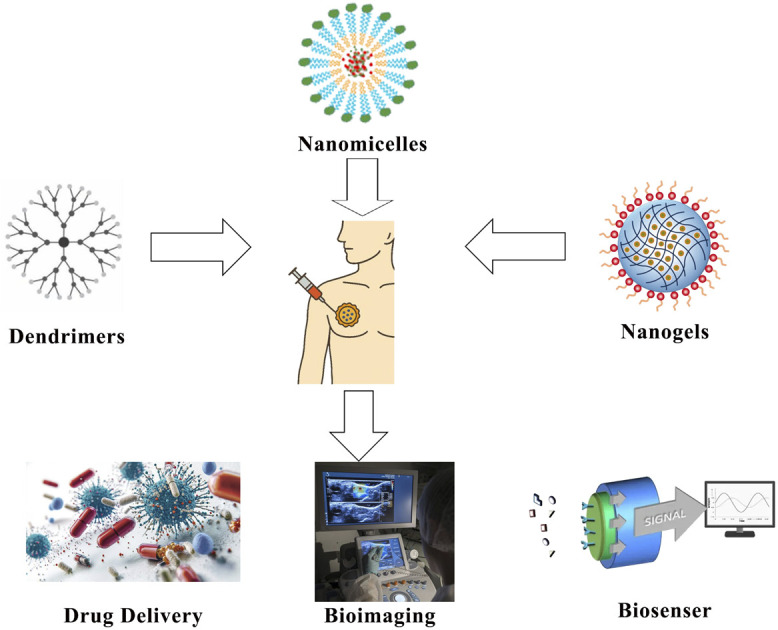

## 1 Introduction

Nanomedicine has been transformed by polymeric nanocarriers—nanomicelles, nanogels, and dendrimers—due to their exceptional ability to deliver therapeutics, enhance diagnostic imaging, and detect biological markers with high precision.^1^ These nanocarriers, ranging from 1 to 200 nm, capitalize on their biocompatibility, structural versatility, and responsive properties to tackle critical barriers in healthcare, such as poor drug solubility, limited tissue access, and low diagnostic sensitivity. By leveraging the enhanced permeability and retention (EPR) effect, nanocarriers achieve selective accumulation in tumors, while their ability to navigate complex biological barriers, such as the blood–brain barrier (BBB), opens new therapeutic avenues.^2,3^ Nanomicelles, formed through the self-organization of amphiphilic block copolymers, solubilize hydrophobic drugs, for example doxorubicin (DOX), and release them in response to stimuli such as pH or redox changes, minimizing systemic toxicity.^2^ Nanogels, composed of hydrophilic crosslinked polymers, encase payloads, such as proteins or nanoparticles, enabling sustained delivery to challenging sites like the central nervous system (CNS).^3^ Dendrimers, with their highly branched architectures, allow precise attachment of drugs or imaging agents, boosting the performance of modalities like MRI and near-infrared (NIR) fluorescence.^4^ In biosensing, their expansive surface areas facilitate the detection of molecules like DNA with exceptional sensitivity.^1^ In contrast to other review articles that often focus on a single application of polymeric nanomaterials, this article integrates three crucial biomedical applications, drug delivery, bioimaging, and biosensing into a single comprehensive system. This comprehensive approach gives scientists a more cohesive platform from which to compare nanomicelles, nanogels, and dendrimers through an assortment of applications and therefore eliminating the use of numerous disparate sources. By consolidating these outcomes, this work addresses a significant gap in the literature and acts as a useful tool for promoting the systematic use and design of polymeric nanomaterials for healthcare applications.

## 2 Nanomicelles

Nanomicelles are colloidal structures, typically ranging from 5 to 100 nm, formed by the self-assembly of amphiphilic monomers or surfactants in aqueous environments. These monomers aggregate above a specific threshold known as the critical micelle concentration (CMC). Each monomer consists of two distinct segments: a hydrophilic head and a hydrophobic tail. The hydrophobic core of the micelle, stabilized by van der Waals forces, encapsulates lipophilic molecules, while the hydrophilic corona forms hydrogen bonds with the surrounding aqueous medium, ensuring steric stability.^5^

### 2.1 Types of nanomicelles

#### 2.1.1 Normal and reverse nanomicelles

Nanomicelles are versatile colloidal structures formed by the self-assembly of amphiphilic molecules, such as surfactants and synthetic block copolymers, in specific solvent environments. In polar solvents, normal nanomicelles form, with hydrophilic segments oriented outward to interact with the solvent and hydrophobic segments clustered in the core to minimize solvent contact. Conversely, in nonpolar solvents, reverse nanomicelles are created, where hydrophobic segments face outward to maximize solvent interaction, and hydrophilic segments reside in the core to avoid contact.^6^ This structural adaptability enables normal nanomicelles to encapsulate insoluble drugs within their hydrophobic core, while reverse nanomicelles can carry soluble drugs in their hydrophilic core.^6^ For biological applications, normal nanomicelles are predominantly used due to their compatibility with aqueous physiological environments.

Nanomicelles have garnered significant attention in nanomedicine for their cost-effectiveness, excellent biocompatibility, straightforward preparation, and therapeutic efficacy.^7^ Compared to conventional micelles, nanomicelles exhibit enhanced thermodynamic stability in physiological solutions, characterized by a lower CMC. This lower CMC ensures greater stability and slower dissociation in the *in vivo* microenvironment, making nanomicelles highly suitable for drug delivery applications.^8^

#### 2.1.2 Surfactant nanomicelles

Nanomicelles can be formed from amphiphilic molecules, such as surfactants and synthetic block copolymers. Surfactants, a key class of amphiphilic molecules, consist of hydrophilic heads and hydrophobic tails, which self-assemble into colloidal dispersions in aqueous environments.^6^ These head groups are categorized as charged (anionic, *e.g.*, sodium dodecyl sulfate; cationic, *e.g.*, dodecyltrimethyl ammonium bromide), dipolar (zwitterionic) (*e.g.*, dioctanoyl phosphatidylcholine, carrying both positive and negative charges), or nonionic (*e.g.*, *n*-dodecyl tetra(ethylene oxide), neutral).^5,9^

**Fig. 1:**
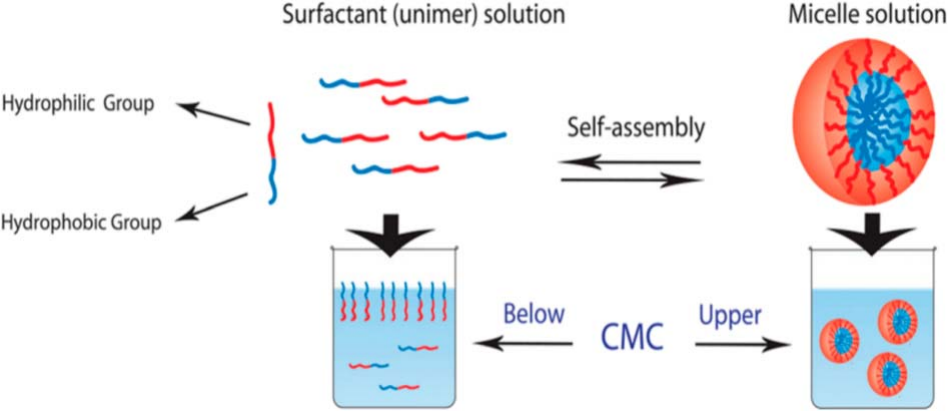
Schematic representation of the equilibrium between unimers (single amphiphilic molecules) and micelles in water. This figure has been reproduced from ref. 6 with permission from *Nanomaterials*, copyright 2020.

At concentrations below the CMC, surfactants align with their hydrophilic heads toward the aqueous solvent, accumulating at air-liquid interface. Above the CMC, surfactants self-assemble into micelles, with hydrophobic tails forming the core and hydrophilic heads interacting with the surrounding medium (Fig. 1).^6^ Low molecular weight surfactants typically exhibit higher CMCs (10^−3^ to 10^−4^ M) compared to polymeric amphiphiles (10^−6^ to 10^−7^ M). Micelles with lower CMCs are preferred for drug delivery systems (DDSs) due to their enhanced stability and reduced sensitivity to dilution, enabling prolonged circulation in the bloodstream.^6^

#### 2.1.3 Polymeric nanomicelles

Polymeric nanomicelles (PNMs), ranging from 10 to 200 nm, are formed from block copolymers with distinct hydrophilic and hydrophobic segments. The hydrophilic outer shell, often composed of poly(ethylene glycol) (PEG) or other polymers (see Section 2.1.3.1), enhances *in vivo* stability and circulation time, while the hydrophobic core encapsulates drugs, controlling their release characteristics.^10,11^

##### 2.1.3.1 Hydrophilic polymers in block copolymers

Hydrophilic polymers are critical components of block copolymers used in polymeric nanomicelles, contributing to their stability and functionality. Commonly used hydrophilic polymers include PEG, poly(*N*-vinyl-2-pyrrolidone) (PVP), poly(2-oxazoline)s (*e.g.*, poly(2-methyl-2-oxazoline) (PMeOx) and poly(2-ethyl-2-oxazoline) (PEtOx)), poly(amino acid)s (*e.g.*, poly(aspartic acid) (P(Asp)), poly(glutamic acid) (P(Glu)), and poly(sarcosine) (PSar)), and polysaccharides (*e.g.*, chitosan, dextran, heparin, hyaluronic acid, and chondroitin sulfate).^6^ Among these, PEG is widely favored due to its excellent physicochemical properties, including high water solubility, low toxicity, chain flexibility, and biocompatibility.^6^ PEG is typically synthesized *via* anionic ring-opening polymerization (ROP) of ethylene oxide, yielding a well-defined polymer with a narrow size distribution.^12^ The chemical versatility of PEG end groups allows the incorporation of reactive moieties for ligand labeling, facilitating conjugation with other micelle components to form targeted block copolymers. These polymers sterically shield the micelle core, minimizing interparticle and blood component interactions, with longer chains (*e.g.*, PEG, poly(ethylene oxide) (PEO)) extending circulation time and reducing reticuloendothelial system (RES) uptake.^6,13^

##### 2.1.3.2 Hydrophobic polymers in block copolymers

The hydrophobic core of polymeric nanomicelles is formed by polymers such as polyesters (*e.g.*, poly(lactic acid) (PLA), poly(ε-caprolactone) (PCL), poly(propylene oxide) (PPO), poly(trimethylene carbonate) (PTMC), and poly(lactic-*co*-glycolic acid) (PLGA)), polyethers (*e.g.*, poly(butylene oxide)), polyanhydrides (*e.g.*, poly(sebacic anhydride) (PSA)), and poly(l-amino acid)s (*e.g.*, poly(l-aspartic acid) (P(Asp)), poly(l-lysine) (P(Lys)), and poly(l-glutamic acid) (P(Glu)).^6^ These hydrophobic polymers are critical for solubilizing hydrophobic drugs and formulating stable polymeric micelles.^6^ Biodegradable polyesters, commonly conjugated with hydrophilic blocks such as PEG to form the micelle's outer shell, create a hydrophobic core that encapsulates drugs. Their *in vivo* degradation minimizes polymer accumulation, reducing the risk of chronic toxicity.^14^ Poly(l-amino acid)s, when conjugated with PEG, can form pH-sensitive nanovesicles, enhancing their utility in targeted drug delivery.^15^ Poly(butylene oxide) also exhibits hydrophobic properties, enabling effective solubilization of hydrophobic drugs when incorporated into block copolymers.^16^

### 2.2 Preparation of polymeric nanomicelles

Polymeric nanomicelles can be prepared using two primary methods: direct dissolution and solvent casting.

#### 2.2.1 Direct dissolution

The direct dissolution, also known as the simple equilibrium method, is commonly employed for block copolymers that are moderately hydrophobic. Representative examples include poloxamers and poly(butyl acrylate) block copolymers with hydrophilic ends, synthesized from one anionic, one cationic, and four nonionic hydrophilic blocks. In this method, both the drug and copolymer are simultaneously dissolved in an aqueous solution, and nanomicelle formation is initiated upon heating. The application of heat facilitates dehydration of the micellar core, driving the self-assembly of nanomicelles. Proper optimization of the drug-to-copolymer ratio and controlled thermal input are critical to achieving stable nanomicelle formation in aqueous environments.^5,17^

#### 2.2.2 Solvent casting

Solvent casting includes four techniques for preparing polymeric nanomicelles: dialysis, oil-in-water (o/w) emulsion, solution casting, and freeze–drying.

##### 2.2.2.1 Dialysis

This method is suitable for non-water-soluble copolymers. The drug and copolymer are dissolved in a water-miscible organic solvent with a high boiling point and placed in a dialysis bag immersed in water for over 12 hours. As the organic solvent gradually evaporates, drug-loaded nanomicelles form. However, this technique may result in drug loss due to low encapsulation efficiency.^6^

##### 2.2.2.2 Oil-in-water (o/w) emulsion

This approach involves dissolving the drug and polymer in a water-immiscible organic solvent mixed with a small volume of water. Evaporation of the organic solvent facilitates physical entrapment of the drug within the hydrophobic core of the nanomicelles.^6^

##### 2.2.2.3 Solution casting

In this method, the drug and polymer are dissolved in an organic solvent to form a transparent solution. After evaporating the solvent under high vacuum, a thin film of drug-loaded nanomicelles is obtained.^6^

##### 2.2.2.4 Freeze–drying

This one-step process involves dissolving the drug and copolymer in a water/*tert*-butanol mixture, followed by lyophilization. *Tert*-butanol forms fine ice crystals that sublime rapidly during freeze–drying, leaving a freeze–dried cake. Rehydration of this cake spontaneously yields drug-loaded nanomicelles.^5^

### 2.3 Polymeric nanomicelles in drug delivery

PNMs have emerged as effective DDSs since their introduction by Kataoka *et al.* in the early 1990s with DOX-loaded micelles for anticancer therapy.^18^ PNMs significantly enhance the water solubility of poorly soluble drugs, increasing solubility by 10- to 5000-fold.^6^ For example, efavirenz, an antiretroviral with low water solubility (approximately 4 mg mL^−1^), achieved a solubility of 34 mg mL^−1^ when encapsulated in PNMs.^6^ These micelles, typically formed from block copolymers with hydrophilic (*e.g.*, PEG) and hydrophobic polyester segments, exhibit high stability and biocompatibility in cancer therapy.^6^ Encapsulated anticancer drugs, such as paclitaxel (PTX) loaded in PCL-*b*-PEG-*b*-PCL micelles (up to 28% loading capacity) or DOX in PEG-*b*-PBLA micelles, demonstrate improved accumulation at target sites, reducing off-target effects on healthy tissues.^19,20^ Additionally, PNMs protect drugs from undesirable interactions and biodegradation *in vivo*, extending drug half-life and enhancing therapeutic efficacy.^21,22^

#### 2.3.1 Micelle biodistribution

The biodistribution of polymeric nanomicelles is governed by their size, shape, and surface charge. Positively charged micelles are more readily taken up by the RES due to enhanced interactions with negatively charged cell membranes, whereas negatively charged micelles reduce cellular uptake and prolong blood circulation time.^6^ Pharmacokinetic studies in mice comparing neutral (Tyr-PEG-*b*-PDLLA) and anionic (Tyr-Glu-PEG-*b*-PDLLA) micelles revealed similar blood clearance kinetics, but anionic micelles exhibited approximately 10-fold lower accumulation in the liver and spleen, attributed to combined steric and electrostatic repulsion.^23^ Spherical micelles (10–30 nm) minimize macrophage capture and are cleared renally, while 30 nm micelles enhance tumor penetration by crossing endothelial junctions, accessing the extravascular extracellular space (EES) for improved anticancer efficacy.^24,25^

#### 2.3.2 Targeted nanomicelles

**Fig. 2:**
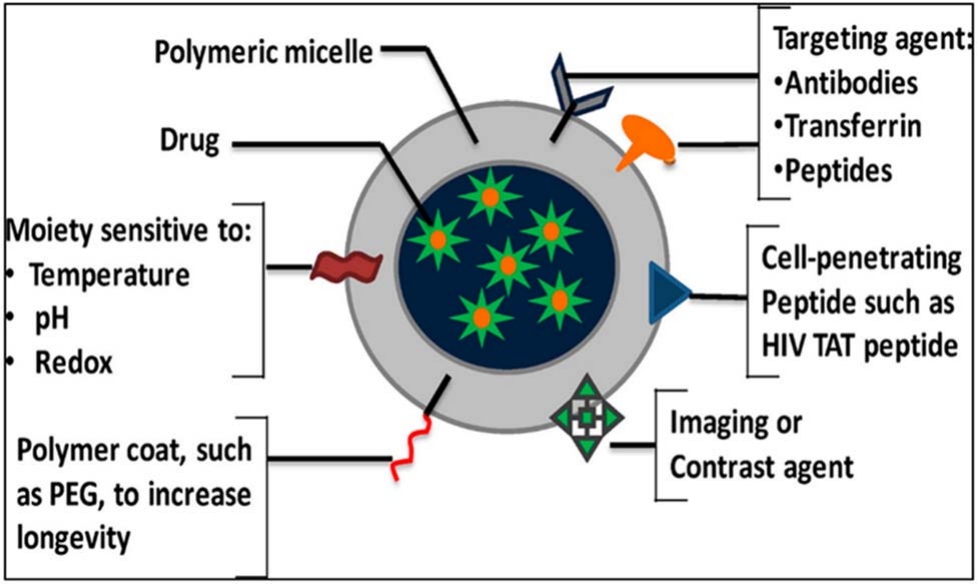
Schematic illustration of polymeric nanomicelles for targeted drug delivery. This figure has been reproduced from ref. 30 with permission from *Eur. J. Pharm. Sci.*, copyright 2016.

Targeted polymeric nanomicelles enhance drug delivery by incorporating ligands, such as antibodies, peptides, or aptamers (Fig. 2), which bind specifically to cell-surface targets or signaling proteins.^5,6^ Folic acid (FA) is a widely used ligand due to its affinity for folate receptors overexpressed on cancer cells. For instance, Park *et al.* developed DOX-loaded PEG-poly(lactide-*co*-glycolide) nanomicelles conjugated with folate for selective cancer cell targeting.^26^ Similarly, Wang *et al.* engineered PTX-loaded PEG-phosphatidylethanolamine (PEG-PE) micelles modified with MCF-7-specific phage fusion proteins to target breast cancer cells.^27^ Additionally, Ahn *et al.* designed antibody fragment-conjugated nanomicelles loaded with platinum to target pancreatic cancer in tumor xenografts.^28^ In 2025, Liu *et al.* designed phospholipid-polyethylene glycol (DSPE-PEG) nanomicelles conjugated with methotrexate (MTX) and combined with amphiphilic copolymers Soluplus and TPGS_1000_ for the treatment of ovarian cancer. Due to high expression of matrix metalloproteinase-2 (MMP-2) in the tumor microenvironment, the hydrophilic shell of the nanomicelle was attached to the PVGLIG peptide. Implementation of the targeting strategy conferred improved stability to the nanomicelles in the circulatory system, enhancing drug accumulation in tumor tissues and ultimately improving therapeutic efficacy.^29^

#### 2.3.3 Stimuli-responsive nanomicelles

**Fig. 3:**
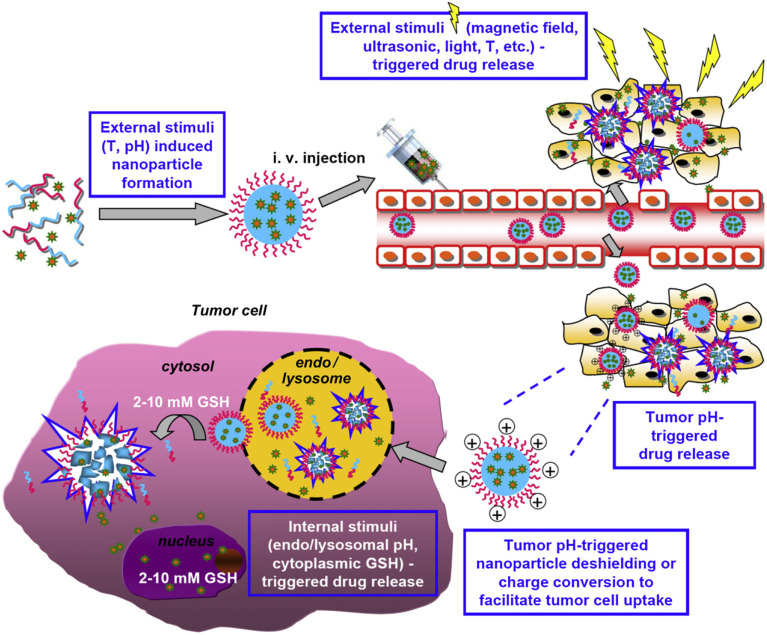
Dual and multi-stimuli-responsive polymeric nanomicelles as advanced controlled drug release systems. This figure has been reproduced from ref. 33 with permission from *Biomaterials*, copyright 2013.

Stimuli-responsive polymeric nanomicelles enable targeted drug release by responding to endogenous or external stimuli, such as pH, temperature, enzyme expression, light, ultrasound, or redox potential, which differ between healthy and diseased tissues (Fig. 2 and 3). These nanomicelles undergo chemical or structural changes to facilitate controlled drug release.^31,32^ Key strategies include: (i) using stimuli such as temperature or pH to drive nanoparticle formation (ii) employing external stimuli (*e.g.*, magnetic fields, ultrasound, light, or temperature); for precise spatial, temporal, and dose-controlled drug release *via* remote activation; (iii) leveraging acidic tumor microenvironments (pH 6.5–7.2) to trigger drug release or reverse nanoparticle shielding, enhancing tumor cell uptake; and (iv) exploiting intracellular conditions, such as low pH in endosomal/lysosomal compartments or high redox potential in the cytoplasm, to optimize drug release within tumor cells.^33^

##### 2.3.3.1 pH-sensitive nanomicelles

###### 2.3.3.1.1 Drug release *via* protonation or deprotonation of polymers

pH-sensitive nanomicelles facilitate controlled drug release through protonation or deprotonation of ionizable polymer groups, triggering hydrophilic–hydrophobic phase transitions. Anionic polymers, such as poly(acrylic acid), poly(methacrylic acid) (PMAA), poly(2-ethyl acrylic acid), and P(Glu), contain carboxylic acid groups that are deprotonated at neutral pH, maintaining micelle stability and hydrophilicity. In acidic environments, these groups become protonated, turning hydrophobic and causing micelle disaggregation to release encapsulated drugs.^6,34^ Cationic polymers with ionizable groups, such as tertiary amines, imidazoles, or pyridines, undergo a hydrophobic-to-hydrophilic transition in acidic conditions, similarly promoting drug release.^6,35^

For example, an amphiphilic block copolymer comprising a hydrophilic PEG segment and a hydrophobic 4-phenyl-1-butanol-modified poly(aspartate) segment was used to encapsulate PTX for anticancer therapy. The unmodified carboxyl groups of poly(aspartate) remain deprotonated at physiological pH, ensuring micelle stability, but become protonated in acidic conditions, triggering PTX release.^36^ In another study, Zhang *et al.* reported methoxy poly(ethylene glycol)-poly(β-amino ester) (mPEG-PAE) nanomicelles encapsulating PAP-1, a selective Kv1.3 channel inhibitor for melanoma treatment. At low pH, poly(β-amino ester) (PAE) segments protonate, destabilizing the micelles and enabling controlled release in the tumor microenvironment. *In vitro*, PAP-1-loaded micelles enhanced cytotoxicity and apoptosis in cancer cells compared to free PAP-1. *In vivo* experiments in a B16F10 melanoma mouse model demonstrated that PAP-1-loaded micelles reduced tumor volume by 94.3%, while free PAP-1 had no significant effect, and minimal toxicity was observed.^37^

###### 2.3.3.1.2 Drug release *via* separation of amphiphilic block micelles

**Fig. 4:**
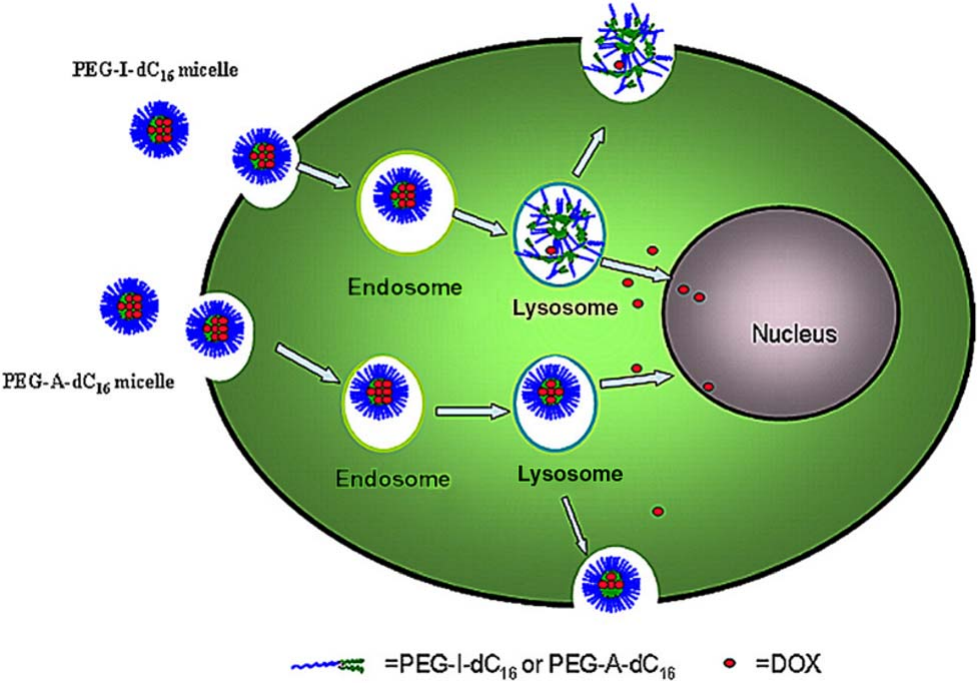
Illustration of self-assembled pH-sensitive micelles for intracellular drug release, demonstrating enhanced drug release into the cytoplasm compared to non-sensitive micelles during endocytosis to exocytosis. This figure has been reproduced from ref. 39 with permission from *J. Pharm. Pharmacol.*, copyright 2016.

pH-sensitive nanomicelles are designed with amphiphilic copolymers linked by pH-responsive bonds to enable targeted drug release in acidic environments. These bonds, stable at physiological pH (7.4), hydrolyze under acidic conditions, causing micelle disassembly and rapid drug release. For instance, Ma *et al.* developed amphiphilic dextran-retinal (DR) micelles by conjugating all-trans retinoic acid to dextran *via* a hydrazone bond. DOX-loaded DR micelles exhibited accelerated drug release in acidic conditions due to cleavage of the acid-labile hydrazone bond.^38^ Similarly, Rongbin *et al.* synthesized pH-sensitive PEG-imine-benzoic-dipalmitate (PEG-I-dC_16_) micelles for efficient DOX delivery, leveraging pH-induced micelle dissociation, as illustrated in Fig. 4.^39^

###### 2.3.3.1.3 Drug release *via* reduced hydrophobicity of polymeric micelles

**Fig. 5:**
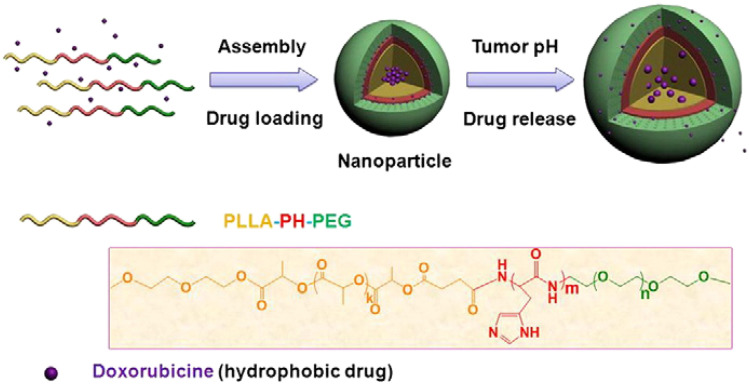
Schematic representation of pH-sensitive drug release from PEG-PH-PLLA nanoparticles. This figure has been reproduced from ref. 40 with permission from *J. Controlled Release*, copyright 2011.

In acidic pH environments, acetal hydrolysis reduces the hydrophobicity of the micelle's hydrophobic core, leading to significant swelling and subsequent drug release. Gu *et al.* developed pH-sensitive nanomicelles from triblock copolymers of poly(ethylene glycol)-poly(l-histidine) (P(His))-poly(l-lactide) (PEG-PH-PLLA), which self-assembled to encapsulate the anticancer drug DOX. The (P(His)) layer undergoes protonation/deprotonation in response to pH changes, triggering micelle swelling and controlled DOX release, as illustrated in Fig. 5.^40^

###### 2.3.3.1.4 Drug release *via* rupture of acid-labile bonds between drug and polymer

In this approach, amphiphilic polymers in nanomicelles remain stable in blood circulation, but acid-sensitive bonds linking the drug to the polymer hydrolyze upon cellular internalization, enabling controlled drug release without micelle depolymerization. Zhong *et al.* developed endosomal pH-sensitive micelles by conjugating PTX to PEG-poly(acrylic acid) (PEG-PAA) copolymers *via* acid-labile acetal bonds, demonstrating effective growth inhibition of human cancer cells.^41^ Similarly, Xu *et al.* designed dual-pH- sensitive nanomicelles for co-delivery of axitinib and DOX, with DOX was conjugated to *N*-(2-hydroxypropyl) methacrylamide (HPMA) *via* an acid-sensitive hydrazone bond, while axitinib was incorporated into the micelle. At the tumor microenvironment (pH 6.4), the benzoic-imine bond hydrolyzed, releasing axitinib. Following endocytosis, the hydrazone bond cleaved in the acidic lysosomal environment (pH 5.0), triggering DOX release.^42^

##### 2.3.3.2 Temperature-sensitive nanomicelles

Temperature-sensitive nanomicelles leverage temperature fluctuations to modulate the CMC and control drug release. Poly(*N*-isopropylacrylamide) (pNIPAM), a widely used temperature-responsive polymer, transitions from hydrophilic to hydrophobic at its lower critical solution temperature (LCST) of approximately 32 °C. Chung *et al.* developed nanomicelles using pNIPAM as the temperature-sensitive hydrophilic shell and poly(butyl methacrylate) (PBMA) as the hydrophobic core. At temperatures above the LCST (*e.g.*, 37 °C), the hydrophilic shell becomes hydrophobic, disrupting interactions with biological components and triggering drug release. For instance, DOX release from pNIPAM-*b*-PBMA nanomicelles reached 90% at 37 °C after 15 hours, compared to only 15% at 30 °C.^43^

In another study, Goodwin *et al.* developed PEG-based micelles with a 2-diazo-1,2-naphthoquinone core, encapsulating a hydrophobic Pt(iv) complex and Fe_3_O_4_ nanoparticles. These nanomicelles demonstrated cytotoxicity against head and neck cancer cells (SQ20B) *in vitro*. By incorporating a near-infrared fluorescent dye and leveraging the magnetic properties of Fe_3_O_4_, the nanomicelles enabled magnetically guided targeting to tumor sites in a live animal xenograft model.^44^ Lin *et al.* designed amphiphilic polymeric mixed micelles with a poly(ε-caprolactone) (PCL) hydrophobic core, triethylene glycol methacrylate (TEGMA) as a temperature-sensitive monomer, disulfide bonds for redox sensitivity, and folic acid for tumor targeting. The micelles exhibited a LCST of >45 °C at neutral pH and 37.6 °C under acidic conditions, ensuring stability during blood circulation but promoting drug release near tumor cells. *In vitro* studies showed that nearly 95% of DOX was released under low pH and high glutathione (GSH) conditions, confirming the temperature- and stimuli-responsive drug release.^45^

##### 2.3.3.3 Light-sensitive nanomicelles

**Fig. 6:**
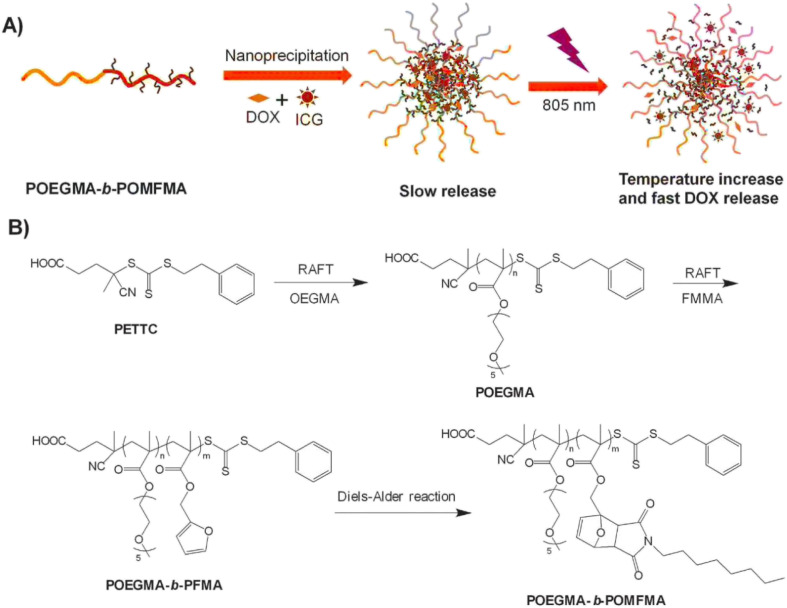
(A) Schematic illustration of ICG and DOX encapsulation and NIR photothermal-triggered drug release. (B) Synthetic route for preparing the block copolymer POEGMA-*b*-PFMA. This figure has been reproduced from ref. 46 with permission from *Macromol. Rapid Commun.*, copyright 2015.

Light-sensitive PNMs enable controlled drug release through irradiation-induced transitions between hydrophobic and hydrophilic states.^6^ These systems utilize exogenous optical stimuli, such as NIR light, to trigger drug release. For example, Goodwin *et al.* demonstrated the release of the fluorescent probe Nile Red from micelles using NIR light.^44^ Similarly, Li *et al.* developed NIR-responsive PNMs composed of poly(oligo(ethylene glycol)methacrylate)-*block*-poly(furfuryl methacrylate) (POEGMA-*b*-PFMA), incorporating indocyanine green (ICG) and DOX. As shown in Fig. 6, NIR irradiation at 805 nm induced a photothermal effect in the micellar core, elevating local temperature and accelerating DOX release for targeted delivery.^46^

In another study, Pourjavadi *et al.* engineered amphiphilic micelles by modifying chitosan biopolymer with poly(acrylamide) and poly(*N*-isopropylacrylamide), complexed with gold nanorods to confer photoresponsiveness. Drug release experiments showed that approximately 38% of PTX was released under NIR irradiation.^47^

##### 2.3.3.4 Ultrasound-responsive nanomicelles

Ultrasound, with frequencies around 20 kHz, is utilized to trigger drug release and enhance cellular uptake in nanomicelle systems. Studies have employed nanomicelles composed of hydrophilic poly(2-methyl-2-oxazoline) combined with hydrophobic polymers such as poly(2-*n*-propyl-2-oxazoline) or poly(2-butyl-2-oxazoline-*co*-2-(3-butenyl)-2-oxazoline).^6^ Marin *et al.* investigated DOX release and intracellular uptake from Pluronic micelles, a common ultrasound-sensitive nanomicelle platform, demonstrating their efficacy in controlled drug delivery.^48^

##### 2.3.3.5 Redox-responsive nanomicelles

The intracellular glutathione concentration in cancer cells is approximately 100-fold higher than extracellular levels, making it an effective target for redox-responsive drug delivery.^6^ Nanomicelles incorporating disulfide bonds are designed to encapsulate drugs, which are released upon bond reduction in the high-glutathione environment of cancer cells. This strategy enables targeted drug release specifically within tumor cells.^6^

##### 2.3.3.6 Multi-stimuli-responsive nanomicelles

Multi-stimuli-responsive nanomicelles, sensitive to pH, temperature, and redox potential, have been developed to enable precise drug delivery.^6^ These nanomicelles feature a hydrophobic core of tetrahydropyran-protected 2-hydroxyethyl methacrylate, which responds to acidic pH changes, and a temperature-sensitive hydrophilic shell of poly(*N*-isopropylacrylamide) (pNIPAM), connected by a redox-sensitive disulfide bond. Above the LCST, the pNIPAM shell transitions from hydrophilic to hydrophobic, promoting micelle assembly by rendering the polymer water-insoluble. In acidic environments, the hydrophobic core becomes hydrophilic, inhibiting assembly. Additionally, elevated redox potential, such as high glutathione levels, reduces the disulfide bond, leading to micelle disassembly and drug release.^49^

A summary of the PNMs discussed above for drug delivery applications is provided in Table 1.

**Table 1:** Application of PNMs in drug delivery

Block copolymer	Drug/vaccine	Indication	Explanation	References
Di-block copolymer of PLGA and PEG conjugated with folate receptors	DOX	Human squamous cell carcinoma cell line of the oral cavity (KB cell) over-expressing folate receptors on the surface	Folate receptor targeted PLGA-PEG micelles entrapping a high loading amount of DOX demonstrated superior cellular uptake over DOX and DOX micelles against a folate-receptor positive cell line	26
Polyethylene glycol phosphatidyl ethanolamine (PEG-PE) micelles composed of MCF-7-specific phage protein	PTX	MCF-7 (tumor cells)	The enhanced anticancer effect was verified by an enhanced apoptosis and reduced tumor cell proliferation following the treatment with the targeted micellar PTX both *in vitro* and *in vivo*	27
Methoxy-poly(ethylene glycol)-*b*-poly(glutamic acid) (MeO-PEG-*b*-P(Glu) and maleimide-PEG-*b*-P(Glu) copolymer (Mal-PEG-*b*-P(Glu) conjugated with antitissue factor antibody (TF)	Platinum drugs	Pancreatic cancer	Fab'-installed platinum-loaded micelles exhibited more than 15-fold increased cellular binding within 1 h and rapid cellular internalization compared to non-targeted micelles, leading to superior *in vitro* cytotoxicity	28
Phospholipid-polyethylene glycol (DSPE-PEG) nanomicelles combined with amphiphilic copolymers soluplus and TPGS_1000_	MTX	SK-OV-3 cells in ovarian cancer	PVGLIG-functionalized nanomicelles: MMP-2-targeted; improved stability and tumor drug deposition; enhanced therapeutic outcomes	29
PEG-4-phenyl-1-butanol modified poly(aspartate)	PTX	Human colorectal cancer cell line HT-29	The carboxyl groups of poly(aspartate) polymer were protonated and could induce the release of PTX at acidic pH (pH-sensitive)	36
PEG-PAE	PAP-1, a potent and selective membrane-permeant Kv1.3 inhibitor	C57BL/6 mice bearing the B16F10 melanoma	The β-amino ester segments of PAE become protonated at low pH, destabilizing the micelles and facilitating controlled drug release in the tumor microenvironment	37
Amphiphilic dextran-retinal (DR) by combining all trans retinoic acid and dextran through a hydrazone bond	DOX	MCF-7 breast cancer cell	pH-sensitive micelles	38
PEG-imine-benzoic-dipalmitate (PEG-I-dC16)	DOX	A549 and HepG2 cancer cells	Acid-sensitive Schiff base groups have been utilized to link PEG and double-stranded C16 chains (pH-sensitive)	39
Poly(ethylene glycol)-poly(l-histidine)-poly(l-lactide) (PEG-PH-PLLA)	DOX	HepG2 liver cancer cells	Poly(l-histidine) as a pH-sensitive was swelled with acidic pH	40
Poly(ethylene glycol)-*b*-poly(acrylic acid) (PEG-PAA) block copolymers	PTX	KB and HeLa cancer cells	PTX is bonded to PAA with acid labile acetal bond (pH-sensitive)	41
Conjugating DOX to *N*-(2 hydroxypropyl) methacrylamide (HPMA) *via* an acidic-sensitive hydrazone group with axitinib, subsequently compressed onto the nanomicelles form	Axitinib (AXI) and DOX	Human non-small cell lung cancer cell line A549 and human umbilical vein endothelial cell line HUVEC	A dual-pH responsive: (DA-CM shows stability at the physiological pH 7.4, and the tumor extracellular pH 6.5 actuates the de-cross-linking of the micelle due to the break of benzoic-imine bond to release AXI, then, at more acidic endolysosomal pH 5.0, DOX was further released due to the hydrolyzed hydrazone linkages)	42
Poly *N*-isopropyl amide (pNIPAMb)_ polybutyl methacrylate (PBMA)	DOX	Bovine aorta endothelial cells (BAECs)	Poly *N*-isopropyl amide as a temperature-sensitive hydrophilic part	43
Poly(ethylene glycol) and 2-diazo-1,2-naphthoquinone with a core–shell structure and encapsulated with a hydrophobic Pt(iv) complex and Fe_3_O_4_ nanoparticles	Platinum	Neck cancer cell (SQ20B)	Temperature-sensitive (taking the advantage of magnetic functionality nanomicelles in the presence of a NIR fluorescent dye)	44
Triethylene glycol methacrylate(TEGMA) – poly(ε-caprolactone) (PCL)	DOX	HeLa cells	*In vitro*, nearly 95% of DOX was released under acidic conditions, demonstrating temperature- and environment-responsive drug delivery	45
Block copolymers, poly(oligo(ethylene glycol) methacrylate)-*block*-poly(furfuryl methacrylate) (POEGMA-*b*-PFMA)	DOX and ICG	Tumor cells	The photothermal effect resulted from 805 nm-NIR irradiation of NIR dye ICG (light-sensitive)	46
Chitosan biopolymer through conjugation with poly(acrylamide) and poly(*N*-isopropyl acrylamide) complexation with the gold nanorods	PTX	MCF7 cells	The drug release experiments displayed that about 38% of PTX drug was released under NIR light irradiation	47
Pluronic micelles	DOX	Promyelocytic leukemia HL-60 cells, ovarian carcinoma drug-sensitive and multidrug-resistant (MDR) cells (A2780 and A2780/ADR, respectively), and breast cancer MCF-7 cells	Ultrasound-sensitive nanomicelles	48

### 2.4 Polymeric nanomicelles in medical imaging

**Fig. 7:**
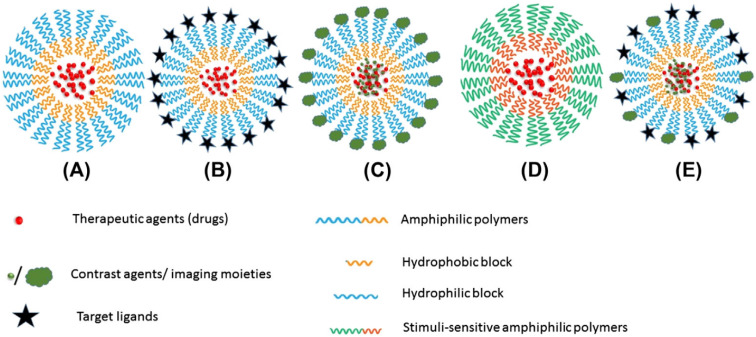
Schematic illustration of (A) polymeric nanomicelle structure, (B) nanomicelle with targeting ligands, (C) nanomicelle with contrast agents or imaging moieties, (D) stimuli-sensitive nanomicelles (temperature-, pH-, light-, or ultrasound-sensitive), and (E) multifunctional nanomicelles integrating targeting ligands, contrast agents, imaging moieties, and therapeutic agents. This figure has been reproduced from ref. 5 with permission from Elsevier, copyright 2017.

PNMs are increasingly utilized in medical imaging and drug delivery due to their unique advantages. These include: (1) nanoscale size, enabling safe intravenous administration with minimal risk of vascular blockage and enhanced tissue accumulation *via* the EPR effect; (2) improved solubility of hydrophobic imaging dyes; (3) high stability and bioavailability; and (4) low toxicity.^5^ PNMs can be engineered as multifunctional platforms, incorporating targeting ligands, contrast agents, imaging moieties, and therapeutic agents, or designed to respond to stimuli such as temperature, pH, light, or ultrasound for precise imaging and drug delivery applications.^5^Fig. 7 illustrates the multifunctional configuration of these nanomicelles, combining targeting, imaging, and therapeutic functionalities.

#### 2.4.1 Computed tomography (CT)

Polymeric nanomicelles enhance CT imaging by encapsulating contrast agents, such as iodine within their hydrophobic core, protecting them from phagocytosis and extending circulation time in the bloodstream. Torchilin *et al.* developed iodine-loaded, long-circulating nanomicelles for improved CT imaging.^5^ Various CT imaging techniques utilizing nanomicelles have demonstrated prolonged blood residence times, as reviewed by Trinh *et al.*^5^

#### 2.4.2 Magnetic resonance imaging (MRI)

Nanomicelles improve MRI by encapsulating paramagnetic metals such as manganese (Mn), gadolinium (Gd), or iron oxide in their hydrophobic core, forming amphiphilic chelating complexes that enhance T1-weighted contrast.^50^ For instance, gadolinium-labeled phosphorescent nanomicelles exhibited superior tumor localization in head and neck tumors.^51^ Song *et al.* developed PEGylated Fe_3_O_4_ nanomicelles that evade RES uptake, enabling effective tumor-targeted MRI diagnosis.^52^ Additionally, Li *et al.* engineered acid-sensitive nanomicelles encapsulating DOX and superparamagnetic iron oxide nanoparticles within poly(ethylene glycol)-*b*-poly(*N*-(*N*′,*N*′-diisopropylaminoethyl) glutamine) (PEG-P(GA-DIP)), surface-modified with FA for targeted drug delivery and enhanced MRI diagnostics in liver cancer cells.^53^ Shen *et al.* recently developed Fe(iii)-coordinated poly(α-amino acid)s (Fe@POS) nanomicelles as a gadolinium-free T_1_ MRI probe. These nanomicelles were synthesized by incorporating Fe(iii) into a poly(3,4-dihydroxy-l-phenylalanine)-*b*-polysarcosine (PDOPA-*b*-PSar, POS) diblock copolymer, achieving high longitudinal relaxivity (*r*_1_ = 5.56 mM^−1^ s^−1^, 20 °C, 3.0 T MR scanner) and prolonged blood circulation. In colorectal cancer and liver metastasis models, Fe@POS provided durable signal enhancement and clearer tumor boundary visualization, highlighting their promise as a safer and more effective alternative to traditional gadolinium-based contrast agents.^54^

#### 2.4.3 Near-infrared fluorescence (NIRF) imaging

**Fig. 8:**
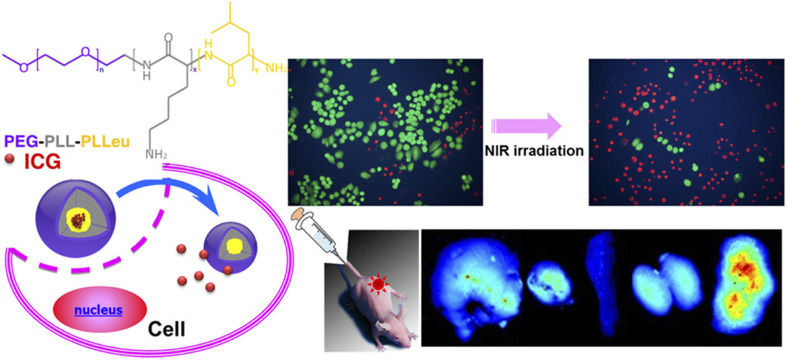
Hybrid polypeptide micelles loaded with ICG for tumor imaging. This figure has been reproduced from ref. 57 with permission from *Biomacromolecules*, copyright 2013.

Near-infrared fluorescence (NIRF) dyes, including phthalocyanines, cyanines, squaraines, 4,4-difluoro-4-bora-3*a*,4*a*-diaza-*s*-indacene (BODIPYs), rhodamine analogs, and porphyrin derivatives, are inherently hydrophobic with limited photostability and weak fluorescence emission *in vivo*. Encapsulation within polymeric nanomicelles addresses these challenges by improving solubility, stability, and fluorescence performance.^5^ For instance, ICG, a hydrophobic carbocyanine dye, can be encapsulated in the hydrophobic core of nanomicelles.^55^ Yang *et al.* developed nanomicelles using monomethoxy poly(ethylene glycol) and alkylamine-grafted poly(l-aspartic acid) to encapsulate carbocyanine dyes for subcutaneous injection, enhancing tumor accumulation, photostability, and signal-to-noise ratio.^56^ Similarly, Wu *et al.* demonstrated that ICG-loaded hybrid polypeptide nanomicelles, composed of poly(ethylene glycol)-*b*-poly(l-lysine)-*b*-poly(l-leucine) (PEG-PLL-PLLeu), significantly improved quantum yield, fluorescence stability, tumor-targeting efficiency, and circulation time, as illustrated in Fig. 8.^57^

**Fig. 9:**
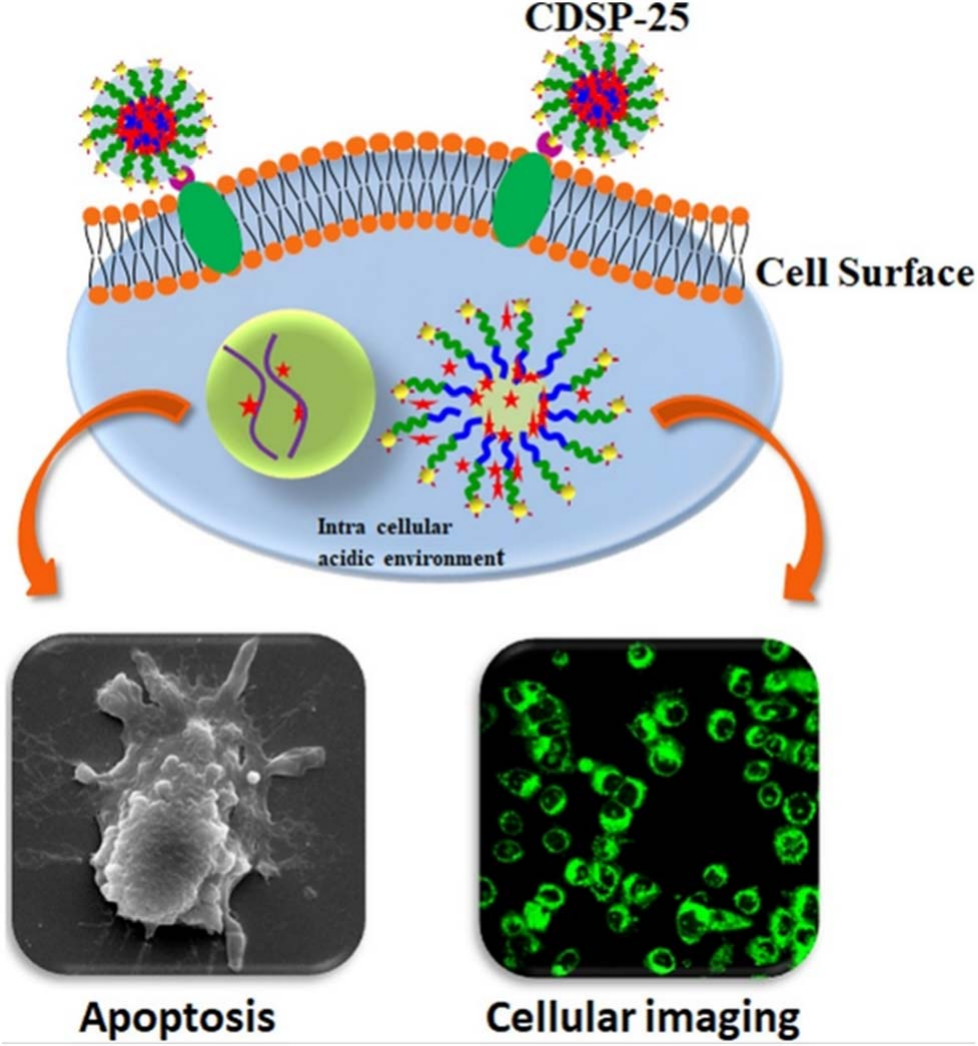
Folic acid-derived carbon dot-functionalized stearic acid-*g*-polyethyleneimine amphiphilic nanomicelles for targeted drug delivery and bioimaging. This figure has been reproduced from ref. 61 with permission from *Colloids Surf., B*, copyright 2021.

Wang *et al.* engineered rod-like PNMs from poly(ethylene oxide)-*block*-poly(sodium acrylate), which exhibited strong NIR emission and stability, effectively staining cancer sites with prolonged retention and selective anticancer efficacy *in vivo*.^58^ Zhang *et al.* synthesized biodegradable poly(ε-caprolactone)-derived PNMs coated with Cy7 fluorescent probes for enhanced imaging.^59^ Kumar *et al.* developed phospholipid nanomicelles loaded with Pt(ii)-tetraphenyltetranaphthoporphyrin (Pt(TPNP)) for NIR imaging.^60^ Sarkar *et al.* created stearic acid-*g*-polyethyleneimine nanomicelles functionalized with FA-derived carbon dots (CDs) for targeted DOX delivery and bioimaging in triple-negative breast cancer (TNBC), leveraging CD fluorescence for effective TNBC imaging, as illustrated in Fig. 9.^61^

In another study, Chen *et al.* designed a NIR-II/photoacoustic (PA) multimodal probe using a CH1055 derivative encapsulated in PEGylated phospholipids (DSPE-PEG5000-NH_2_) and conjugated with an *anti*-EGFR Affibody, enabling precise targeting of EGFR-positive tumors in an FTC-133 mouse model with superior NIR-II and PA signals.^62^ In 2025, Chepurna *et al.* developed core–shell nanoparticles (∼250 nm) consisting of a ∼30 nm polystyrene core and a thermosensitive shell of poly(*N*-isopropylacrylamide-*co*-acrylamide) [poly(NIPAM-*co*-AA)], loaded with near-infrared-shortwave infrared (NIR-SWIR) dyes (3782SL, HPPH) for near-infrared fluorescence imaging and photodynamic therapy. The nanomicelles shrink above their LCST (∼39 °C), enhancing both the fluorescence of the encapsulated dyes and singlet oxygen generation. *In vitro*, heated cancer cells exhibited a 2–3-fold increase in fluorescence, while *in vivo* studies demonstrated thermally triggered signal enhancement following localized NIR laser heating. These findings underscore the potential of this system for temperature-controlled imaging and therapy.^63^

A summary of the PNMs discussed above for medical imaging applications is presented in Table 2.

**Table 2:** Application of PNMs in medical imaging

Nano carriers	Detection	Imaging technique	References
Gadolinium-labeled phosphorescent polymeric phospholipid-based nanomicelles	Detection of head and neck tumor	MRI	51
PEGylated Fe_3_O_4_ nanomicelles	4T1 breast cancer in mouse model	MRI	52
DOX and superparamagnetic iron oxide nanoparticles encapsulated in poly(ethylene glycol)-*b*-poly(*N*-(*N*′,*N*′-diisopropylaminoethyl) glutamine) (PEG-P(GA-DIP)) nanomicelles and surface-modified with FA	Liver cancer cells	MRI	53
Fe(iii)-coordinated poly(α-amino acid)s (Fe@POS) nanomicelles by poly(3,4-dihydroxy-l-phenylalanine)-*b*-polysarcosine (PDOPA-*b*-PSar, POS) diblock copolymer	Colorectal cancer	MRI	54
PEG and alkylamine-grafted poly(l-aspartic acid) carbocyanine dye-loaded nanomicelles	Hypervascular and hypovascular tumors	NIRF imaging	56
ICG – loaded hybrid polypeptide nanomicelles poly(ethylene glycol)-*b*-poly(l-lysine)-*b*-poly(l-leucine) (PEG-PLL-PLLeu)	Human non-small cell lung carcinoma (H460) cells	NIRF imaging and photothermal effect	57
Rod-like nanomicelles based on poly(ethylene oxide)-*block*-poly(sodium acrylate) complexed with rhodium(i)	Cancer cells (4T1 cell line in nude mice)	Near-infrared phosphorescence imaging	58
Poly(ethylene glycol) and polycaprolactone coated with a fluorescent probe (Cy7) on the surface	Cancer cells	Fluorescent imaging	59
NIR phosphorescent molecules of Pt(ii)-tetraphenyltetranaphthoporphyrin [Pt(TPNP)] encapsulated into phospholipid nanomicelles	Pancreatic tumors	Near-IR phosphorescent	60
Stearic acid-*g*-polyethyleneimine amphiphilic nanomicelles functionalized with FA-derived carbon dots (CDs)	TNBC	Fluorescence imaging and DOX delivery	61
Donor–acceptor chromophore based nanoparticle (DAP) as a dual-modal image contrast agent encapsulated into amphiphilic PEGylated phospholipids (DSPE-PEG5000-NH_2_)	EGFR-positive tumors in FTC-133 subcutaneous mice model	Fluorescence and photoacoustic imaging	62
Core–shell nanoparticles with a polystyrene core and a thermosensitive shell of a co-polymer of *N*-isopropylacrylamide and acrylamide [poly(NIPAM-*co*-AA)] loaded with NIR dye 3782SL or photosensitizer HPPH	Lewis lung carcinoma cells	NIR fluorescence imaging and photodynamic therapy	63

### 2.5 Polymeric nanomicelles in biosensors

**Fig. 10:**
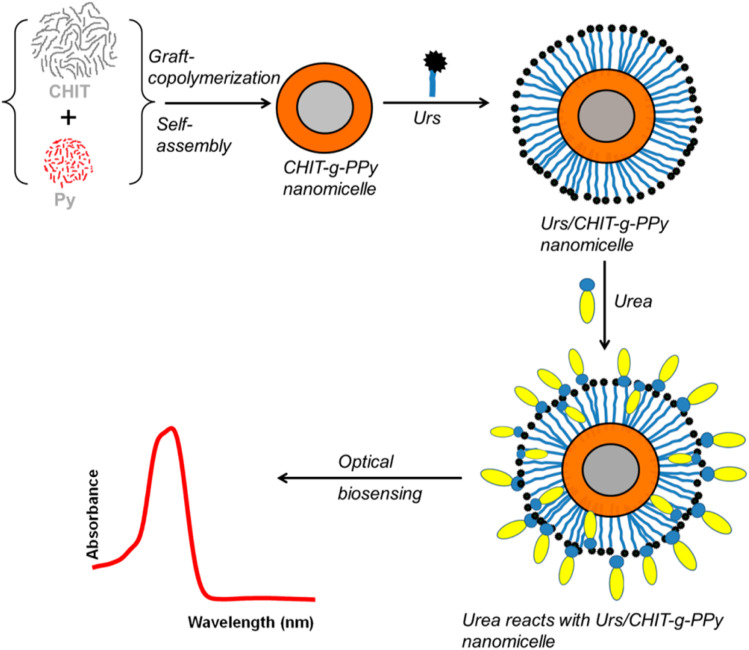
Schematic representation of assembled CHIT-*g*-PPy/urease nanostructures and the optical sensing process. This figure has been reproduced from ref. 65 with permission from *Ind. Eng. Chem. Res.*, copyright 2014.

PNMs are promising platforms for biosensor applications due to their high surface-to-volume ratio and functional properties. These micelles enhance sensitivity, stability, enzyme reusability, and loading capacity while reducing production costs and measurement times in enzymatic biosensors.^64^ Shukla *et al.* developed an optical urea sensor using chitosan-*g*-polypyrrole (CHIT-*g*-PPy) spherical nanomicelles, synthesized *via* covalent bonding and conjugated with urease enzyme (CHIT-*g*-PPy/Urs). As shown in Fig. 10, the sensor quantifies urea through the enzymatic hydrolysis of urea to ammonia, which reacts with Nessler's reagent (K_2_HgI_4_) to form a colored complex (NH_2_Hg_2_I_3_). Absorbance at 385 nm enables accurate urea detection and evaluation of the sensor's analytical performance.^65^

**Fig. 11:**
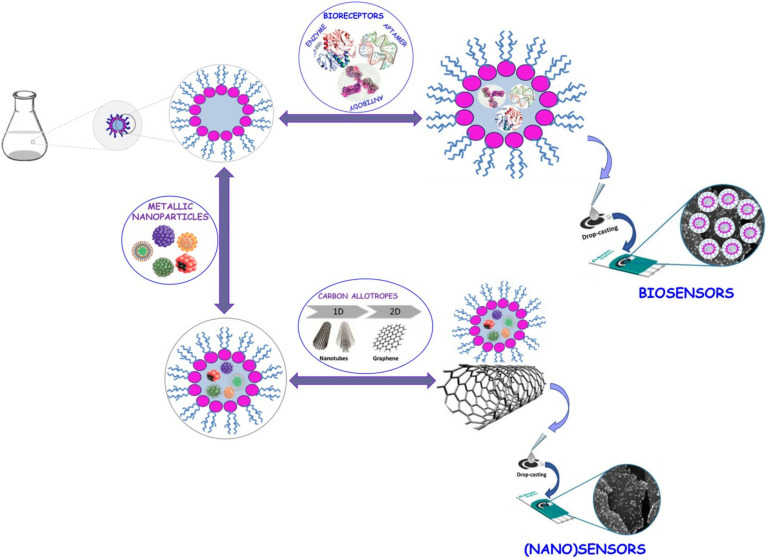
Schematic representation of nano(bio)sensor design using reverse micelles. This figure has been reproduced from ref. 66 with permission from *Processes*, copyright 2021.

Normal and reverse nanomicelles are utilized to prepare enzyme-immobilized metallic and quantum dot (QD) nanoparticles, enhancing enzymatic activity and durability for biosensor applications.^65^ Reverse micelles, in particular, serve as effective platforms for entrapping nanomaterials and bioreceptors, as illustrated in Fig. 11.^66^

Merkoçi *et al.* developed QD-based biosensors using cadmium sulfide (CdS), lead sulfide (PbS), and zinc sulfide (ZnS) nanoparticles synthesized *via* a reverse micelle method with AOT/*n*-heptane water-in-oil microemulsion, incorporating cadmium, lead, or zinc nitrate and sodium sulfide. These QDs were conjugated with thiolated oligonucleotides and used with streptavidin-coated magnetic beads for sensitive DNA detection.^67^ Fernandes *et al.* created a biosensor for hydroquinone detection by immobilizing peroxidase on gold electrodes coated with well-dispersed gold nanoparticles (AuNPs) stabilized by a zwitterionic surfactant. This biosensor enabled electrochemical detection of hydroquinone in skin-brightening creams at a low potential of 0.09 V *vs.* Ag/AgCl, with a detection limit of 0.188 μmol L^−1.^^68^ Similarly, Moreira Ferreira *et al.* developed an electrochemical sensor using rhodium nanoparticles stabilized with 3-(1-tetradecyl-3-imidazolium) propane-sulfonate surfactant to coat a glassy carbon electrode. This sensor exhibited enhanced electrical conductivity and reduced electron transfer resistance, enabling reliable detection of *p*-coumaric acid in a cellulose matrix with a detection limit of 0.6 μmol L^−1^ and high reproducibility.^69^

Table 3 shows a summary of the PNMs discussed above for biosensing applications.

**Table 3:** Application of PNMs in biosensors

Method	Detection	Explanation	References
Urease enzyme immobilized on chitosan-*g*-polypyrrole (CHIT-*g*-PPy) polymeric nanomicelles	Detection of urea, a key indexes in diagnosing liver and kidney disorders	This optical urea biosensor showed a linear response to urea concentrations ranging from 0.01 to 30 mM and exhibited a sensitivity of 0.25 μM with a response time of 12 s	65
QDs to an AOT/*n*-heptane water-in-oil microemulsion (reverse micelles)	Detection of DNA	The detection of DNA was achieved by using streptavidin-coated magnetic beads and CdS QDs	67
Immobilization of peroxidase onto gold electrodes stabilized with zwitterionic surfactant (reverse micelles)	Determination of hydroquinone	With a detection limit of 0.188 μmol L^−1^	68
Coated of surface of a glassy carbon electrode with rhodium nanoparticles, stabilized with the 3-(1-tetradecyl-3-imidazolium) propane-sulfonate surfactant (reverse micelles)	Determination of *p*-coumaric acid in a cellulose matrix	At a detection limit of 0.6 μmol L^−1^	69

## 3. Nanogels

Nanogels are three-dimensional, hydrophilic polymer networks, ranging from 1 to 200 nm in size, formed through physical or chemical crosslinking. These nanoscale structures can absorb substantial amounts of water or biological fluids while retaining structural integrity.^70,71^ Their small size, high surface area, biocompatibility, and tunable properties make them ideal for biomedical applications, including drug delivery, medical imaging, and biosensing. Nanogels can efficiently encapsulate small-molecule drugs, biomacromolecules, and inorganic nanoparticles, enabling their use in therapeutic and imaging applications for various medical conditions.^72,73^

### 3.1 Types of nanogels

#### 3.1.1 Natural polymer nanogels

Natural polymer nanogels, derived from biocompatible and non-immunogenic materials such as chitosan, alginate, hyaluronic acid, or pullulan, are widely used in pharmaceutical applications.^74,75^ Pullulan-based nanogels, for instance, can be chemically modified to form complexes with proteins, drugs, or DNA, and to coat cells, liposomes, or particles, making them ideal for targeted drug delivery and tissue engineering.^76^

#### 3.1.2 Synthetic polymer nanogels

Synthetic nanogels, synthesized from polymers such as PEG, poloxamers, polyacrylates, or polypeptides, offer precise control over size, structure, and stimuli-responsive properties.^77^ PEG-based nanogels, prepared *via* emulsion polymerization or click chemistry, are hydrophilic, biocompatible, and often engineered for pH- or temperature-sensitive drug release, enabling tailored pharmacokinetics for advanced biomedical applications.^77,78^

#### 3.1.3 Chemically crosslinked nanogels

Chemically crosslinked nanogels are stabilized by covalent bonds, such as amide, disulfide, Schiff base, or photo-induced linkages, providing high structural stability.^71,79,80^ These nanogels are synthesized through methods like emulsion polymerization, reversible addition–fragmentation chain transfer (RAFT), or photo-induced crosslinking. Photo-responsive nanogels, activated by light, are particularly effective for targeted drug delivery, offering versatility and precision in therapeutic applications.^3,81,82^

#### 3.1.4 Physically crosslinked nanogels

Physically crosslinked nanogels are formed through noncovalent interactions, including hydrogen bonding, hydrophobic interactions, or electrostatic forces, resulting in lower stability but simpler preparation.^83^ These nanogels are well-suited for applications such as transdermal drug delivery, where temporary stability and stimuli-responsive behavior enhance functionality.^71^

#### 3.1.5 Structural types of nanogels

Nanogels are classified into several structural types, including hollow, multilayered, core–shell, and hairy variants, each designed for specific functions such as enhanced surface area or improved environmental interaction.^84^ Nanohydrogels, composed of nanoscale hydrogels, provide biocompatibility and controlled drug release, while nanoorganogels encapsulate oily substances, forming micelle-like systems.^81,85^ Micellar nanogels, formed *via* self-assembly of hydrophobic and hydrophilic moieties, feature a core–shell structure that enables simple physical entrapment of drugs.^86^ Hybrid nanogels, consisting of nanogel particles dispersed within an organic or inorganic matrix, exhibit high efficiency in targeted drug release.^87^ Liposome-modified nanogels serve as advanced carriers for transdermal delivery, responding to stimuli such as pH or temperature.^71^ These diverse structures allow nanogels to encapsulate a wide range of therapeutic agents, including small molecules, anti-neoplastic drugs, and insulin, through physical, chemical, or electrostatic interactions with the nanogel network.^86^

### 3.2 Preparation of nanogels

Nanogel preparation methods are tailored to the polymer type and intended application, focusing on controlling size, structure, and stimuli-responsive properties.

#### 3.2.1 Emulsion-based methods

**Fig. 12:**
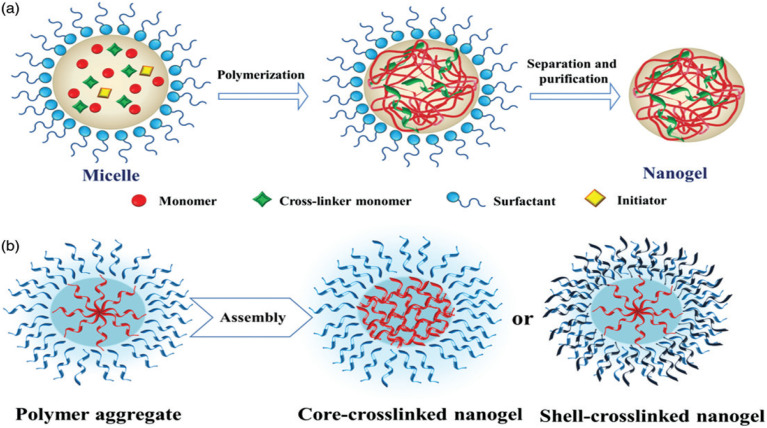
Preparation of nanogels *via* copolymerization in a colloidal environment: (a) direct polymerization of monomers; (b) assembly of a polymer precursor. This figure has been reproduced from ref. 75 with permission from *Drug Delivery*, copyright 2017.

**Fig. 13:**
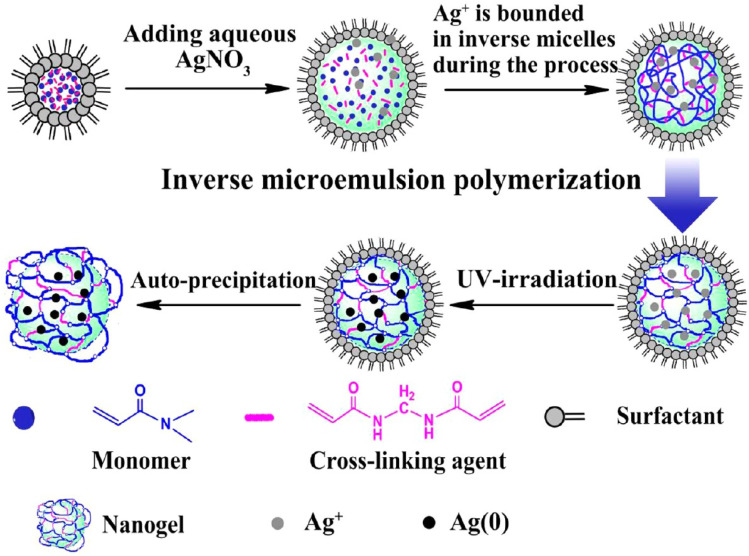
Process of inverse microemulsion for Ag-poly(*N*,*N*-dimethylacrylamide) (Ag/PDMAA) nanogel synthesis. This figure has been reproduced from ref. 91 with permission from *Langmuir*, copyright 2013.

Emulsion-based methods, particularly water-in-oil emulsions, are widely used to synthesize nanogels with precise size control. These methods involve emulsifying aqueous biopolymer droplets in a continuous oil phase using oil-soluble surfactants, followed by crosslinking with water-soluble agents. Techniques include inverse miniemulsion, membrane emulsification, and reverse micellar methods.^88,89^ Emulsion polymerization, conducted in the presence of surfactants, is a common approach to produce nanogels with well-defined sizes through direct monomer polymerization or assembly of polymer precursors.^90^The preparation of nanogels *via* copolymerization in a colloidal environment is illustrated in Fig. 12, showing both direct polymerization of monomers and assembly of a polymer precursor. Additionally, the synthesis of Ag-poly(*N*,*N*-dimethylacrylamide) (Ag/PDMAA) nanogels using the inverse microemulsion method is depicted in Fig. 13, enabling controlled nanogel formation with uniform particle size and morphology.

#### 3.2.2 Self-assembly

**Fig. 14:**
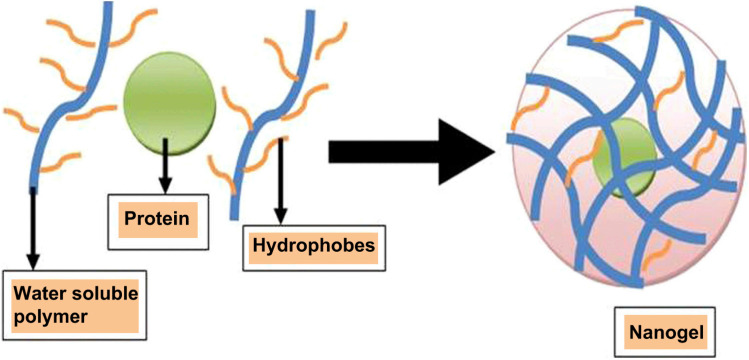
Aggregation of hydrophobically modified cholesterol-pullulan in the presence of insulin, resulting in nanogels with entrapped protein. This figure has been reproduced from ref. 88 with permission from *Artif. Cells, Nanomed., Biotechnol.*, copyright 2016.

Self-assembly is a spontaneous, reversible process where molecular units organize into thermodynamically stable, well-defined supramolecular structures with minimal defects. This method, driven by weak non-covalent interactions such as hydrogen bonds, van der Waals forces, coulombic interactions, and hydrophobic effects, offers adaptability, cost-effectiveness, and robust structures due to its occurrence at thermodynamic equilibrium.^92,93^ In polyelectrolyte-based nanogels, self-assembly leverages interactions between oppositely charged electrolytes, achieving high loading efficiency for therapeutic agents.^94^ For example, as shown in Fig. 14, hydrophobically modified cholesterol-pullulan polymers aggregate in the presence of insulin, forming nanogels that efficiently entrap the protein.^88^

### 3.3 Nanogels in drug delivery

**Fig. 15:**
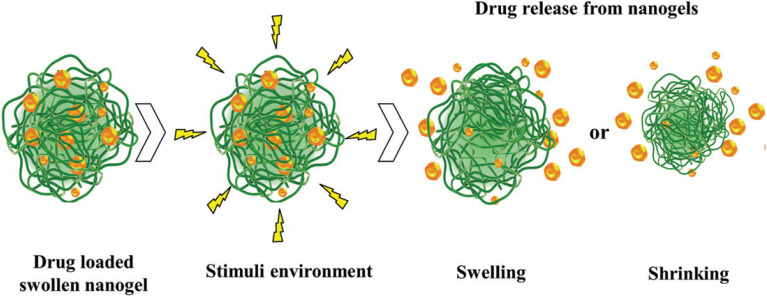
Schematic diagram of the light-responsive mechanism involved in drug release from nanogels. This figure has been reproduced from ref. 75 with permission from *Drug Delivery*, copyright 2017.

Nanogels are versatile drug delivery vehicles capable of encapsulating diverse payloads, such as small molecules, proteins, peptides, nucleic acids, and inorganic nanoparticles, while protecting them from degradation.^95^ Drugs are incorporated through physical entrapment, electrostatic interactions, or covalent bonding, with release modulated by nanogel swelling or stimuli-responsive mechanisms.^83^ Conventional nanogels swell *via* water absorption, enabling sustained drug release, while stimuli-responsive nanogels, triggered by pH, temperature, enzymes, light, or redox conditions, provide precise control over drug release through swelling or deswelling (Fig. 15).^83^ Nanogels can be administered *via* various routes, including injection, oral, pulmonary, nasal, parenteral, and intraocular, and their ability to cross cellular barriers, such as the BBB *via* endocytic pathways, makes them ideal for targeted therapies, including brain tumor treatment and tissue engineering.^71,96,97^ Their capacity to deliver drugs to the CNS underscores their potential as advanced nanocarriers.^86^

#### 3.3.1 Nanogels in cancer therapy

**Fig. 16:**
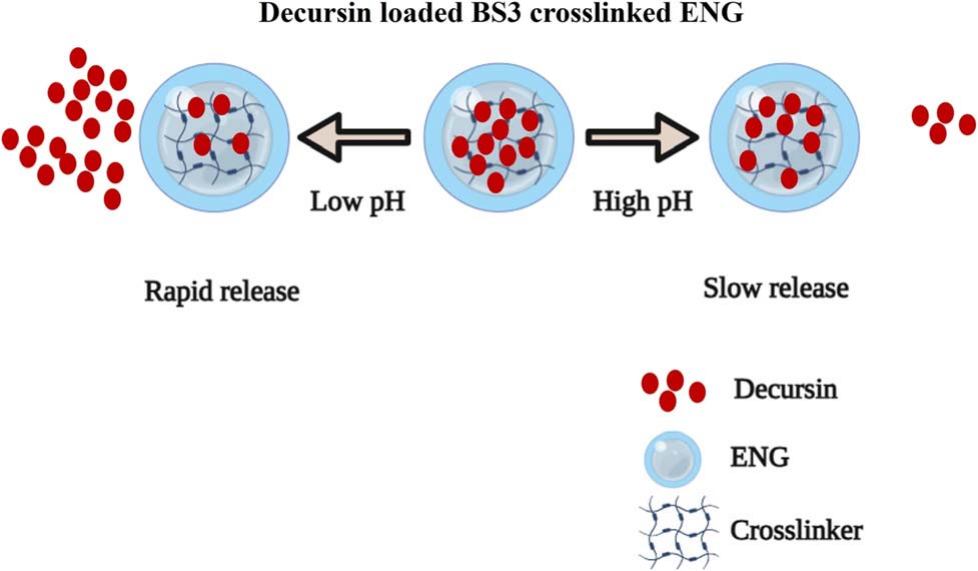
Release of decursin (DEC) from elastin-based nanogel (ENG) in response to pH changes. This figure has been reproduced from ref. 98 with permission from *Sci. Rep.*, copyright 2024.

Nanogels are effective carriers for delivering anticancer drugs, such as temozolomide, cisplatin, 5-fluorouracil, heparin, and doxorubicin, to tumors in organs such as the liver, breast, prostate, and lung, improving therapeutic outcomes while minimizing damage to healthy tissues. For instance, an elastin-based nanogel (ENG) was synthesized *via* inverse miniemulsion and crosslinked with bis(sulfosuccinimidyl) suberate (BS3) to encapsulate decursin (DEC) for targeted prostate cancer (PCa) therapy. As shown in Fig. 16, the ENG exhibits pH-responsive behavior, with amide group ionization inducing swelling and increased flexibility in acidic tumor microenvironments. This weakens DEC-ENG interactions and enlarges pore sizes, accelerating DEC release at lower pH compared to neutral or higher pH conditions, thereby enhancing DEC solubility and therapeutic efficacy for PCa treatment.^98^

**Fig. 17:**
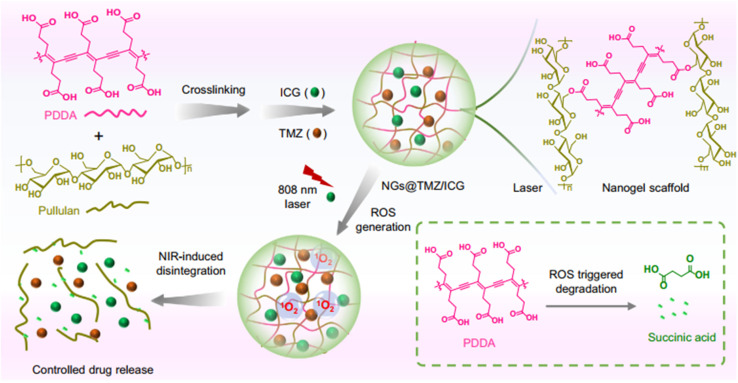
Schematic illustration of biomimetic nanogel synthesis and NIR-induced disintegration. This figure has been reproduced from ref. 99 with permission from *Nat. Commun.*, copyright 2022.

Zhang *et al.* developed a biomimetic nanogel system by crosslinking pullulan with poly(deca-4,6-diynedioic acid) (PDDA), encapsulating ICG, an FDA-approved NIR photosensitizer, and temozolomide (TMZ), a standard chemotherapeutic for glioblastoma (GBM). These nanogels, coated with ApoE peptide-modified erythrocyte membranes and loaded with TMZ and ICG (ARNGs@TMZ/ICG), enhance blood circulation and tumor targeting. The coating ensures stability under physiological conditions, promoting efficient accumulation in GBM lesions following intravenous administration. Upon NIR irradiation at peak tumor accumulation, ICG generates reactive oxygen species (ROS), disrupting the nanogels and triggering rapid release of TMZ and ICG. This facilitates extravasation and deep tumor penetration, amplifying the combined photodynamic-chemotherapeutic effect (Fig. 17). In orthotopic GBM mouse models, this NIR-activatable nanogel system significantly improved therapeutic outcomes, highlighting its potential for brain tumor therapy.^99^

#### 3.3.2 Transdermal drug delivery

Nanogels enhance topical and transdermal delivery of anti-inflammatory drugs, such as spantide II, ketoprofen, and aceclofenac, for conditions such as psoriasis and dermatitis, improving skin permeation while minimizing side effects associated with oral administration.^84,100^ Shah *et al.* developed nanogels incorporating surface-modified bilayered nanoparticles (NPs) made from poly(lactide-*co*-glycolic acid) and chitosan, with oleic acid modification (NPSO) to enhance skin contact time and hydration *via* an occlusive effect. These nanogels, formulated with hydroxypropyl methylcellulose (HPMC) and carbopol for optimal viscosity, significantly improved the skin permeation of spantide II and ketoprofen into deeper skin layers.^101^ In another study, Toyoda *et al.* utilized iontophoresis to deliver antigen peptide-loaded nanogels, which accumulated in the epidermis, activated Langerhans cells, and suppressed tumor growth, demonstrating promising potential for transdermal immunotherapy.^102^ In 2025 study, Nait Bachir *et al.* engineered gelatin/xanthan-aldehyde nanogels *via* Schiff base chemistry to improve ibuprofen transdermal delivery. The optimized nanogels (∼180 nm) exhibited high encapsulation efficiency (∼94%) and globular, porous morphology. *In vitro* release tests showed sustained dissolution, while *ex vivo* Franz cell assays confirmed enhanced skin permeation compared with free ibuprofen. *In vivo* evaluation using ibuprofen-loaded nanogel patches demonstrated strong anti-inflammatory activity, achieved 82% edema inhibition, outperforming commercial gels, highlighting nanogels as a promising platform for transdermal drug delivery.^103^

#### 3.3.3 Gene therapy

**Fig. 18:**
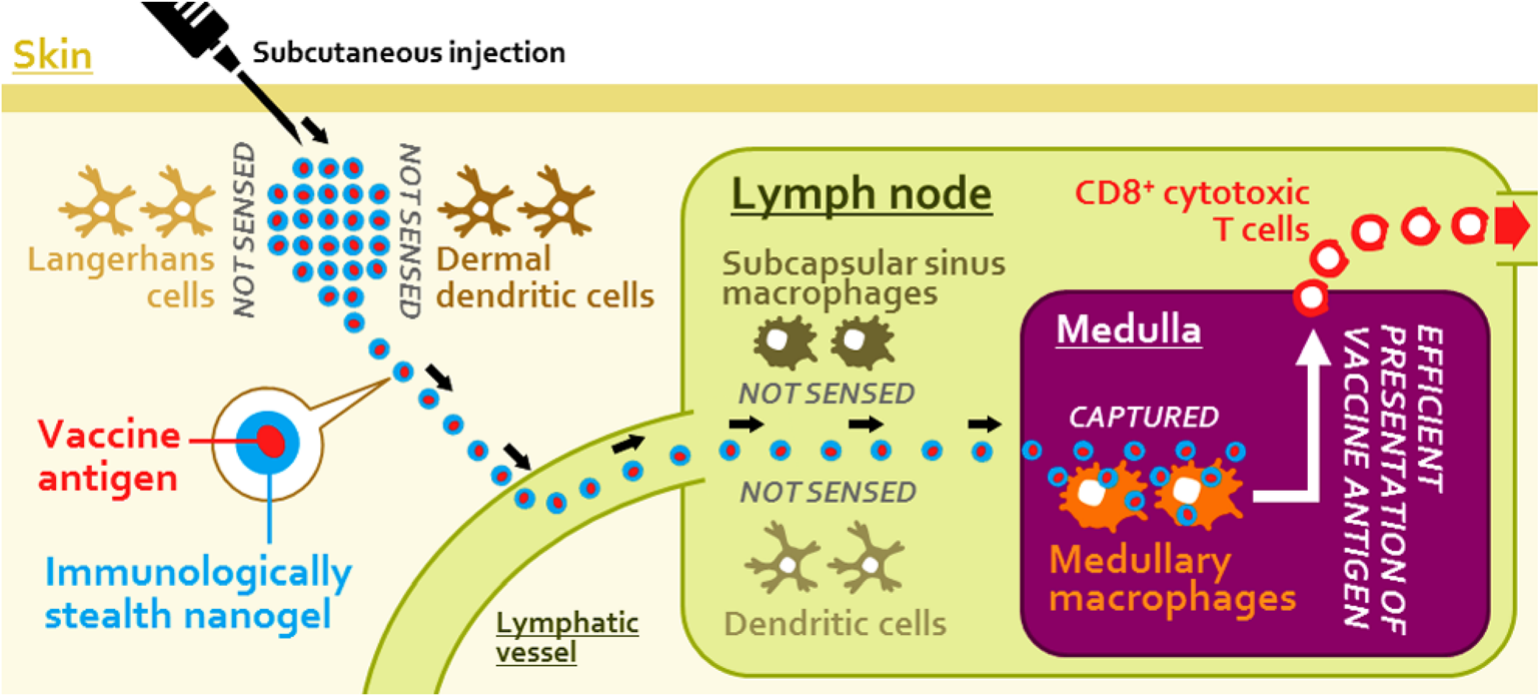
Nanogel-based immunologically stealth vaccine targeting macrophages in the medulla of lymph nodes, inducing potent antitumor immunity. This figure has been reproduced from ref. 106 with permission from *ACS Nano*, copyright 2014.

Nanogels are effective carriers for gene therapy, delivering therapeutic DNA or RNA sequences to treat genetic diseases. Tetrahedral DNA-based nanogels protect small interfering RNA (siRNA), facilitating gene silencing for targeted genetic therapies.^104^ Polysaccharide-based self-assembled nanogels have been utilized for tumor-associated antigen delivery, enhancing immunotherapy.^105^ For instance, Muraoka *et al.* developed cholesteryl pullulan (CHP) nanogels to deliver long peptide antigens (LPAs) to draining lymph nodes. By engaging medullary macrophages and eliciting a robust CD8^+^ T cell response, these nanogels demonstrated significant immunotherapeutic efficacy in animal models, highlighting their potential for antitumor immunity, as illustrated in Fig. 18.^106^

In a recent study, Gao *et al.* developed PEGylated cationic nanogels with a core of poly(*N*-(4-vinylbenzyl)-*N*,*N*-dimethylamine) (PVBDMA) crosslinked *via* a glutathione-responsive linker for siRNA delivery. Using a regulated PEGylation strategy, they minimized protein corona formation and enhanced cellular uptake. The nanogels were loaded with PLK1-specific siRNA (siPLK1), achieving efficient endocytosis, endosomal escape, and cytoplasmic release. These nanogels demonstrated significant gene silencing *in vitro* and tumor growth inhibition in an orthotopic ovarian tumor mouse model, highlighting their potential for siRNA-based gene therapy.^107^

#### 3.3.4 Vaginal drug delivery

Nanogels are promising carriers for vaginal drug delivery, enabling targeted treatment of conditions such as vaginal infections by incorporating antibacterial agents to reduce discomfort and abnormal discharge.^108^ Ghorbanali *et al.* developed nanocomposite hydrogel rings composed of polyacrylamide, sodium carboxymethyl cellulose, and montmorillonite nanoparticles, molded into a ring-shaped aluminum mold for controlled drug release. *In vitro* studies using methylene blue as a hydrophilic model drug demonstrated that these nanocomposite rings reduced the burst release effect by over twofold and sustained drug release for 15 days in a vaginal fluid simulant (pH ∼4.2, 37 °C). Additionally, varying montmorillonite concentrations in the hydrogels exhibited different antibacterial activities against *Escherichia coli*, highlighting their potential for effective vaginal drug delivery.^109^

#### 3.3.5 Diabetes treatment

Injectable nanogels offer a promising approach for diabetes management by enabling self-regulated insulin delivery in response to hyperglycemic conditions. These nanogels are designed to sense changes in blood glucose levels, releasing insulin accordingly.^86^ Mudassir *et al.* developed a pH-sensitive nanogel based on poly(methyl methacrylate) (MMA) and itaconic acid (IA) for oral insulin delivery. This system enhances insulin bioavailability and patient compliance by overcoming gastrointestinal epithelial barriers, providing an alternative to injectable insulin.^110^

#### 3.3.6 Ophthalmic drug delivery

Nanogels are an effective platform for ophthalmic drug delivery, facilitating prolonged drug retention at target sites. Klingner *et al.* synthesized a pH-sensitive nanogel composed of poly(vinylpyrrolidone) and poly(acrylic acid) (PAA)*via* γ-radiation-induced polymerization. This nanogel encapsulated pilocarpine, a drug used to treat various eye conditions, achieving extended drug retention and improved therapeutic efficacy.^111^ Recently, Bruno *et al.* developed chitosan nanogels for ocular delivery of FA. The nanogels showed spherical morphology, stability after lyophilization, and confirmed FA – chitosan interactions *via* FTIR, DSC, and TGA. *In vitro* studies demonstrated sustained FA release (Higuchi model) compared to free FA. *Ex vivo* rabbit cornea tests showed encapsulated FA permeated 2.6× slower than free FA, supporting controlled release and prolonged ocular retention. This system enhances bioavailability and therapeutic efficacy while reducing dosing frequency for ophthalmic use.^112^

Table 4 shows a summary of the nanogels discussed above for drug delivery applications.

**Table 4:** Applications of nanogels in drug delivery

Nanocarrier	Drug/agent	Indication	Explanation	References
ENG with bis(sulfosuccinimidyl) suberate (BS3) crosslinking	DEC	Castration-resistant prostate cancer (CRPC)	pH-sensitive release mechanism enhances DEC solubility and release in acidic tumor microenvironments	98
Pullulan/poly(deca-4,6-diynedioic acid) (PDDA) nanogels coated with ApoE peptide-modified erythrocyte membranes	TMZ and ICG	GBM	NIR-activatable nanogels release TMZ and ICG upon ROS generation, improving GBM therapy *via* photodynamic-chemotherapeutic effects	99
Skin permeating nanogel system (SPN) containing surface modified polymeric bilayered nanoparticles along with a gelling agent	Two anti-inflammatory drugs, spantide II (SP) and ketoprofen (KP)	Delivery of SP and KP to the deeper skin layers for treatment of various skin inflammatory disorders	These nanogels enable percutaneous delivery of SP and KP to deeper skin layers, treating various skin inflammatory disorders	101
Cancer antigen gp-100 peptide KVPRNQDWL-loaded PEG-modified nanogels	Cancer antigen gp-100 peptide KVPRNQDWL (anti-cancer vaccination)	Langerhans cells	Iontophoresis of peptide-loaded nanogels activated Langerhans cells and suppressed tumor growth	102
Gelatin/xanthan-aldehyde nanogels	Ibuprofen an anti-inflammatory drugs	Wistar rat abdominal skin	*In vivo* evaluation using ibuprofen-loaded nanogel patches demonstrated strong anti-inflammatory activity, with up to 82% edema inhibition—far superior to commercial reference gels	103
Immunologically inert nanoparticulate hydrogel (nanogel) of cholesteryl pullulan (CHP)	Long peptide antigen (LPA)	Medullary macrophages in the lymph node	Delivers LPA to lymph nodes, provoking CD8^+^ T cell response for immunotherapeutic effect	106
PEGylated cationic nanogels with a core of poly(*N*-(4-vinylbenzyl)-*N*,*N*-dimethylamine) (PVBDMA) crosslinked *via* a glutathione-responsive linker	PLK1-specific siRNA (siPLK1)	Downregulating polo-like kinase 1 (PLK1) for treating ovarian cancer	These nanogels demonstrated significant gene silencing *in vitro* and tumor growth inhibition in an orthotopic ovarian tumor mouse model, highlighting their potential for siRNA-based gene therapy	107
Hydrogel rings based on polyacrylamide-sodium carboxymethyl cellulose-montmorillonite nanoparticles	Methylene blue	*Escherichia coli* and vaginal infections	These hydrogel rings exhibit varying antibacterial activities against *Escherichia coli* and vaginal infections based on montmorillonite concentrations	109
pH-sensitive polyelectrolyte methyl methacrylate (MMA)/itaconic acid (IA) nanogels	Insulin	Diabetes	pH-sensitive nanogel enhances oral insulin delivery, improving bioavailability across gastrointestinal barriers	110
pH-sensitive poly(vinylpyrrolidone)-poly(acrylic acid) (PVP/PAA)	Pilocarpine	Treat various eye conditions	Maintain satisfactory extended retention of the drug at the target site	111
Chitosan nanogels	FA	Rabbit corneas	*Ex vivo* rabbit cornea tests showed encapsulated FA permeated 2.6× slower than free FA, supporting controlled release and prolonged ocular retention	112

### 3.4 Nanogels in medical imaging

Nanogels are promising platforms for medical imaging due to their small size, biocompatibility, and ability to encapsulate contrast agents, enhancing tumor accumulation through the EPR effect. Their stability and targeting capabilities make them suitable for various imaging modalities.^113^

#### 3.4.1 Magnetic resonance imaging (MRI)

**Fig. 19:**
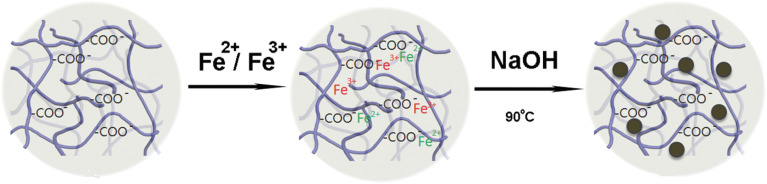
Schematic of the synthetic route for hybrid Fe_3_O_4_-PAA nanogels. This figure has been reproduced from ref. 115 with permission from *J. Biomed. Nanotechnol.*, copyright 2015.

Delivering MRI contrast agents selectively to target tissues remains a challenge for many clinical formulations. Podgórna *et al.* developed gadolinium alginate nanogels for glioma diagnosis, demonstrating targeted and sensitive imaging for personalized theranostics in oncology.^114^ Similarly, Chen *et al.* synthesized poly(acrylic acid) (PAA)-derived nanogels encapsulating superparamagnetic Fe_3_O_4_ nanoparticles (11–16 nm) *via in situ* co-precipitation (Fig. 19). These hybrid nanogels exhibited high biocompatibility, efficient internalization in human neuroblastoma SH-SY5Y cells, and excellent T2-weighted MRI contrast, with enhanced sensitivity at tumor sites.^115^

Hu *et al.* developed a manganese-loaded pH-responsive DNA hydrogel (M-TDH) for thyroid tumor-targeted MRI. The nanogel, synthesized *via* rolling circle amplification and Mn-induced biomineralization, incorporated a thyroglobulin (Tg) aptamer for tumor-specific recognition. *In vitro*, M-TDH demonstrated pH-triggered Mn^2+^ release and high affinity toward Tg-expressing thyroid cancer cells. *In vivo*, NCG mice bearing both thyroid (TPC1) and breast (MDA-MB-231) tumor xenografts, M-TDH enabled prolonged T1-weighted signal enhancement in thyroid tumors (up to 48 h), outperforming MnCl_2_ and Gd-DTPA.^116^

#### 3.4.2 Fluorescence imaging

**Fig. 20:**
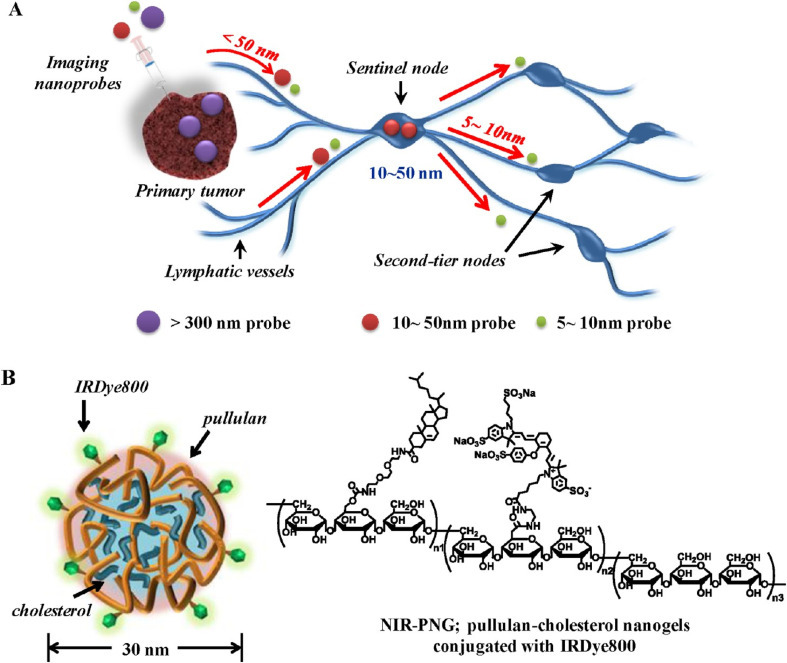
Schematic illustration of NIR-PNG nanoprobes optimized for SLN mapping. (A) SLN mapping using nanoscale imaging probes, with 10–50 nm nanoprobes showing rapid SLN uptake and retention. (B) Chemical structure of NIR-PNG based on pullulan-cholesterol nanogels conjugated with IRDye800. This figure has been reproduced from ref. 119 with permission from *ACS Nano*, copyright 2012.

NIR probes, such as QDs, AuNPs, and organic dyes such as cyanines and BODIPYs, often suffer from poor water solubility and rapid *in vivo* degradation, limiting their clinical utility.^117^ Encapsulation or conjugation with hydrophilic nanogels enhances their aqueous stability, photostability, and suitability for NIR fluorescence imaging.^117,118^ For instance, Cao *et al.* developed polyurethane nanogels encapsulating 8-hydroxypyrene-1-carbaldehyde (HPC), a pH-sensitive dye, enabling fluorescence imaging of H_2_O_2_-induced cytosolic acidosis.^118^ Noh *et al.* improved sentinel lymph node (SLN) imaging by conjugating IRDye800 to 30 nm cholesterol-modified pullulan nanogels, as depicted in Fig. 20, achieving a sixfold higher SLN signal intensity in mice 30 minutes post-intradermal injection compared to free dyes, due to enhanced photostability and pharmacokinetics.^119^

Jayakumar *et al.* synthesized QD-incorporated chitin nanogels for theranostic applications, maintaining luminescence for over 24 hours post-internalization, demonstrating their potential for prolonged optical and NIR imaging.^120^ Mok *et al.* developed hyaluronidase (HAdase)-activatable NIR nanogels using ICG-conjugated hyaluronic acid (HA). The NIR signal, quenched until activated by HAdase or low pH, enabled tumor-specific imaging in mice for up to three days, highlighting their diagnostic potential.^121–124^ Jiang *et al.* engineered pH- and thermo-sensitive nanogels encapsulating Fe_3_O_4_ and conjugated with Cy5.5-labeled lactoferrin for dual MRI and fluorescence imaging of brain gliomas. At pH 6.4, the nanogels' LCST decreased, causing them to shrink at physiological temperature and enhancing tumor cell uptake. In rat models, these nanogels showed significantly higher glioma uptake 48 hours post-injection, demonstrating precise brain tumor imaging.^125^

Sun *et al.* developed tumor-targeting, redox-responsive nanogels (TRNGs) based on hyaluronic acid–lipoic acid conjugates for cytochrome c (CC) delivery. *In vitro* and *in vivo* fluorescence imaging confirmed tumor-specific accumulation in CD44-positive A549 tumor cells and lung tumor-bearing mice, *via* CD44-mediated endocytosis and subsequent cytosolic release. TRNGs enabled therapeutic protein delivery and real-time visualization, leveraging the EPR effect, with minimal systemic toxicity.^126^

#### 3.4.3 Positron emission tomography (PET)

Nanogels enhance positron emission tomography (PET) imaging by incorporating metal-chelating crosslinkers for stable radionuclide encapsulation. Lux *et al.* developed a PET tracer by integrating ^64^Cu, with a 12.7 hour half-life, and 1,4,7-triazacyclononane-1,4,7-triacetic acid (NOTA) crosslinkers into polyacrylamide (PAAm) nanogels. These nanogels retained 94% of copper after 48 hours in serum and demonstrated high uptake in 4T1 mouse breast tumors, achieving a tumor/muscle signal ratio exceeding nine at 48 hours post-injection. This formulation effectively imaged both primary tumors and metastases, underscoring its potential as a robust PET tracer for oncology.^127^

#### 3.4.4 Photoacoustic (PA) imaging

Photoacoustic (PA) imaging, leveraging optical and ultrasound principles, benefits from nanogel-based systems for combined imaging and therapy.^128,129^ Shi *et al.* developed γ-polyglutamic acid (γ-PGA) nanogels crosslinked with cystamine dihydrochloride (Cys) *via* EDC coupling, loaded with polyaniline (PANI) as a photothermal agent. With an average hydrodynamic size of 689 nm and excellent colloidal stability, these γ-PGA/Cys@PANI nanogels exhibited a linear increase in PA signal intensity with concentration, confirming their efficacy as contrast agents for PA imaging and their potential for integrated imaging-guided photothermal therapy.^130^

Table 5 shows a summary of the nanogels discussed above for medical imaging applications.

**Table 5:** Applications of nanogels in medical imaging

Nanocarrier	Detection	Imaging technique	References
Gadolinium alginate nanogels	Glioma	MRI	114
Hybrid Fe_3_O_4_-PAA nanogels	Murine hepatic carcinoma H22cells	MRI	115
DNA hydroge loaded with Mn^2+^ and incorporated with thyroglobulin (Tg) aptamer	Thyroid (TPC1) and breast (MDA-MB-231) tumor	MRI	116
Polyurethane nanogels encapsulating 8-hydroxypyrene-1-carbaldehyde (HPC), a pH-sensitive dye	Imaging of H_2_O_2_-induced cytosolic acidosis	Fluorescence imaging	118
IRDye800-conjugated cholesterol-modified pullulan nanogels	SLN	NIR fluorescence imaging	119
QD-incorporated chitin nanogels	L929 (mouse fibroblast cell line), NIH-3T3 (mouse embryonic fibroblast cell line), KB (oral cancer cell line), VERO cells (kidney epithelial cell line of the African green monkey), MCF-7 (human breast cancer cell line) and PC3 (prostate cancer cell line)	NIR fluorescence imaging	120
ICG encapsulated HA nanogels	Detection of HAdase activity *in vivo* (HAdase-associated diseases: specific cancers and lymph nodes)	NIR fluorescence imaging	123 and 124
Encapsulating iron oxide (Fe_3_O_4_) within *p* (NIPAM-*co*-AA) (PNA) nanogels and conjugated with Cy5.5-labeled lactoferrin	Brain glioma	MRI and fluorescence imaging	125
Lipoic acid incorporated in methoxy poly(ethylene glycol) and diethylenetriamine-modified hyaluronic acid (HA-*g*-mPEG/Deta-*c*-LA)	CD44-positive A549 cells (lung tumor-bearing mice)	Fluorescence imaging	126
Polyacrylamide (PAAm) nanogels with ^64^Cu and NOTA crosslinker	4T1 breast tumors	PET/MRI imaging	127
γ-Polyglutamic acid (γ-PGA) nanogels loaded with polyaniline (PANI), a photothermal agent and crosslinked with cystamine dihydrochloride (Cys)	The 4T1 tumor-bearing mice	PA and PTT imaging	130

### 3.5 Nanogels in biosensors

**Fig. 21:**
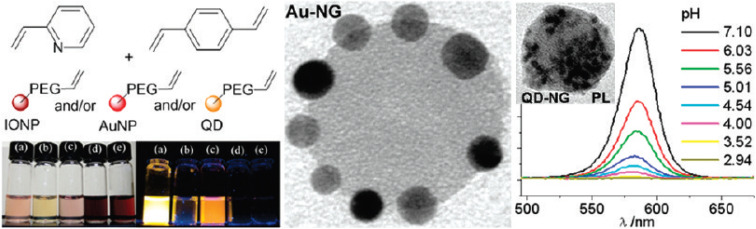
(A) Schematic of allyl-PEG-capped inorganic NPs, including magnetic iron oxide NPs (IONPs). (B) TEM micrographs of 10 nm AuNPs (10 nm Au-NG) at pH 7. (C) Photoluminescence of QD-NGs at various pH levels, showing bright fluorescence at pH 7 and quenching at pH 3. This figure has been reproduced from ref. 134 with permission from *Nano Lett.*, copyright 2011.

Nanogels are highly effective in biosensing applications due to their hydrophilicity, small size, and rapid responsiveness to stimuli such as pH, temperature, or glucose levels. They serve as versatile platforms in three key roles: encapsulating detector molecules, acting as stimuli-responsive materials to enhance detection, and functioning as sensory membranes.^131,132^ Nanogels can encapsulate inorganic and organic nanoparticles (NPs), including quantum dots (QDs), magnetic NPs, and metallic NPs, to improve biosensor sensitivity, optical properties, and detection range.^133^ Riedinger *et al.* developed pH-responsive poly(2-vinylpyridine-*co*-divinylbenzene) nanogels encapsulating allyl-PEG-capped inorganic NPs, such as magnetic iron oxide NPs, CdSe/ZnS QDs, and AuNPs. The photoluminescence (PL) of QD-loaded nanogels (QD-NGs) was bright at pH 7 but quenched at lower pH, becoming completely and irreversibly lost at pH 3 (Fig. 21). This pH-dependent PL behavior enables QD-NGs to function as optical pH sensors in the pH 4–7 range, suitable for tracking cargo delivery within cellular compartments, particularly in acidic environments below pH 5, where nanogel swelling facilitates detection.^134^

The chitosan-poly(methacrylic acid) (PMAA)-CdSe hybrid nanogel serves as a multifunctional biosensor platform. This nanogel integrates a pH-responsive chitosan-PMAA matrix crosslinked with CdSe QDs. The semi-interpenetrating chitosan and PMAA network undergoes pH-induced volume phase transitions, while homogeneously dispersed CdSe QDs convert these changes into optical signals, enabling effective pH sensing.^135^ The composite structure enhances component interactions, improving biosensor functionality. Recent studies demonstrated pH-induced expulsion of PMAA chains from these nanogels over 32 hours, exceeding the 20 hour timeframe for macromolecular colloids to accumulate in tumors *via* blood circulation.^136^ This dynamic response, driven by pH-induced charge accumulation within the weak polyelectrolyte nanogel, highlights its potential for sensitive and responsive biosensing applications.^137–140^

Polymer-inorganic hybrid nanogels with temperature-responsive properties are increasingly significant in biomaterials science and bionanomedicine, particularly for biosensing applications. Zhu *et al.* developed a hybrid nanogel system combining Bi_2_O_3_ QDs with poly(vinyl alcohol) (PVA) nanogel networks. These Bi_2_O_3_@PVA nanogels respond to temperature changes in the physiological range (37–40 °C), converting environmental temperature disruptions into sensitive fluorescent signals. They also enable dark-field and fluorescence dual-modal imaging in mouse melanoma B16F10 cells and regulate the release of TMZ, a model anticancer drug.^141^ Similarly, Li *et al.* engineered poly(*N*-isopropylacrylamide) (PNIPAM)-based nanogels as dual fluorescent sensors for temperature and Hg^2+^ ions, leveraging thermo-induced PNIPAM collapse to enhance detection sensitivity. They also developed a PNIPAM nanogel sensor for Cr^3+^ detection, covalently labeled with rhodamine B urea derivatives (P(NIPAM-*co*-RhBUA)), which exhibited a ∼61-fold fluorescence intensity increase at 45 °C, significantly improving Cr^3+^ detection sensitivity and selectivity.^142^

**Fig. 22:**
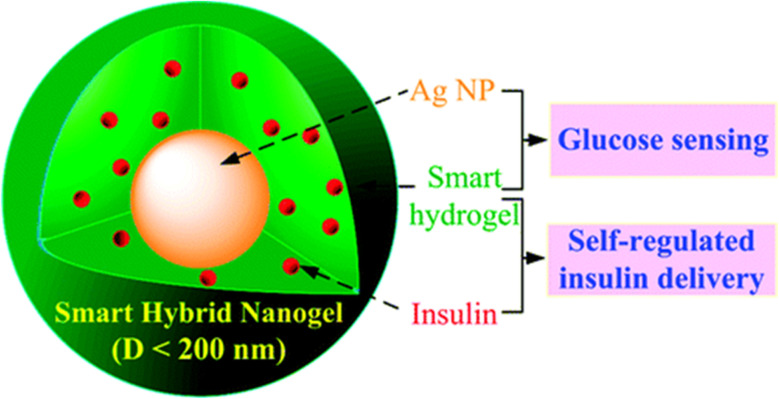
Schematic illustration of smart hybrid nanogels integrating optical glucose detection and self-regulated insulin release at physiological pH and temperature. This figure has been reproduced from ref. 143 with permission from *ACS Nano*, copyright 2010.

Core–shell nanogels, notable for their biocompatibility, biostability, and streamlined synthesis, derive responsive properties primarily from their encapsulated cores. For example, silver nanoparticles (AgNPs) form a fluorescent core encased in a poly(4-vinylphenylboronic acid-*co*-2-(dimethylamino)ethyl acrylate) [p(VPBA-DMAEA)] gel shell. The AgNPs provide robust, non-bleaching fluorescence, while the p(VPBA-DMAEA) shell, with boronic acid-based ligands, enables glucose sensing. Glucose-induced swelling or shrinking of the shell translates concentrations into optical signals, facilitating controlled insulin release at physiological pH and temperature (Fig. 22).^143^

Robby *et al.* developed a coenzyme A (CoA)-responsive polyallylamine-MnO_2_-polymer dot nanogel-coated electrochemical sensor for detecting osteoarthritis (OA) genetic models using chondrocytes. The sensor responded to CoA *via* a redox reaction (MnO_2_ → Mn^2+^), leading to conductivity and fluorescence changes. Coupled with a bluetooth wireless device, the system enabled real-time smartphone monitoring, supporting its role as a sensitive, portable OA diagnostic platform.^144^

A summary of the nanogels discussed above for biosensors applications is provided in Table 6.

**Table 6:** Applications of nanogels in biosensors

Method	Detection	Explanation	References
Poly(2-vinylpyridine-*co*-divinylbenzene) nanogels with magnetic iron oxide (IONPs), CdSe/ZnS QDs, and AuNPs encapsulated (fluorescence sensors)	pH change sensors	The bright fluorescence of the QD-NG exhibited at pH 7 was quenched when the pH was lowered and it was lost completely and irreversibly at pH 3. Association with pH-responsive nanogels significantly affect their local pH environment, altering photoluminescence (PL) decay	134
Chitosan-poly(methacrylic acid) (PMAA)-CdSe hybrid nanogel (fluorescence sensors)	pH variations across physiological conditions	Multifunctional system for simultaneous optical pH-sensing, tumor cell imaging, and pH-regulated delivery of anticancer drug	135
Bi_2_O_3_ QDs with poly(vinyl alcohol) nanogel (fluorescence sensors)	Temperature change sensors	Convert the disruptions in homeostasis of environmental temperature into high-sensitive fluorescent signals	141
Thermoresponsive poly(*N*-isopropylacrylamide) (PNIPAM)-based nanogels with 1,8-naphthalimide-based fluorescent monomer (NPTUA) and boronic acid-based ligands (fluorescence sensors)	Temperature and Hg^2+^ ions	Thermo-induced collapse enhances Hg^2+^ detection sensitivity in a dual fluorescent sensor	142
Hybrid nanogels made of AgNP cores covered by a copolymer gel shell of poly(4-vinylphenylboronic acid-*co*-2-(dimethylamino)ethyl acrylate) [p(VPBA-DMAEA)] (fluorescence sensors)	Optical detection of glucose	Glucose-induced swelling/shrinking of boronic acid-based shell converts concentration into optical signals for insulin delivery regulation	143
Coenzyme A (CoA) – responsive polyallylamine-MnO_2_-polymer dot nanogel-coated electrochemical sensor	Osteoarthritis (OA) genetic models	The sensor responded to CoA *via* a redox reaction (MnO_2_ → Mn^2+^), leading to conductivity and fluorescence changes	144

## 4. Dendrimers

Dendrimers are hyperbranched polymers with precisely controlled three-dimensional architectures, allowing tailored mass, size, shape, and surface chemistry.^145^ The term “dendrimer,” derived from the Greek word “dendron” meaning tree or branch, was first introduced by Buhleier in 1978 and further developed by Tomalia in the 1980s.^146^ Their unique structure includes internal cavities and open conformations (in low-generation dendrimers), enabling encapsulation of hydrophobic drug molecules. Additionally, the high density of surface functional groups facilitates conjugation with biomolecules or contrast agents. These properties make dendrimers highly suitable for applications in drug delivery, medical imaging, and biosensing.^145,147^

### 4.1 Types of dendrimers

Dendrimers encompass over 100 distinct structures, with poly(amidoamine) (PAMAM), poly(propylene imine) (PPI), polyamide, polyether, polyester, and phosphorus-based dendrimers being the most prominent families.^146^

#### 4.1.1 Poly(amidoamine) (PAMAM) dendrimers

PAMAM dendrimers, first developed by Tomalia, are among the most widely studied dendritic structures, characterized by an ethylenediamine or ammonia core and amidoamine branches.^148,149^ Their ellipsoidal or spheroidal morphology, combined with numerous functional terminal groups and internal cavities, provides high reactivity and solubility, making them ideal for biomedical applications.^149^ PAMAM dendrimers enable controlled drug release, enhanced tumor accumulation, and reduced toxicity compared to other nanocarriers.^150^ Surface modification with PEG minimizes non-specific interactions with plasma proteins and phagocytosis, prolonging blood circulation time and improving therapeutic efficacy.^150^ However, higher-generation PAMAM dendrimers (G6 and above) may exhibit increased toxicity and higher production costs.^146^

#### 4.1.2 Poly(propylene imine) (PPI) dendrimers

Poly(propylene imine) (PPI) dendrimers are amine-terminated, hyperbranched macromolecules featuring a butylenediamine core and propylene imine branches.^148,149^ These dendrimers possess polyalkylamine structures with primary amine terminal groups and internal voids containing tertiary tris-propyleneamines, providing high reactivity and versatility for molecular encapsulation.^149^ Commercially available up to the fifth generation (G5), PPI dendrimers are widely utilized in biology and material science due to their tunable surface chemistry and ability to encapsulate guest molecules within their internal cavities.^149^

#### 4.1.3 Liquid crystalline (LC) dendrimers

Liquid crystalline (LC) dendrimers are specialized macromolecules synthesized from mesogenic monomers, such as mesogen-functionalized carbosilane dendrimers.^148,149^ These dendrimers form thermotropic liquid crystalline phases or mesophases, driven by rod-like (calamitic) or disk-like (discotic) mesogenic units, which confer unique self-assembling and anisotropic properties suitable for advanced applications.^148^

#### 4.1.4 Core–shell (tecto) dendrimers

Core–shell tecto-dendrimers are sophisticated polymeric nanostructures formed by the controlled covalent attachment of dendrimer units, featuring a central core dendrimer surrounded by peripheral dendrimers.^148,149^ This highly ordered architecture allows each component to perform specific functions, making tecto-dendrimers ideal for smart therapeutic nanodevices.^149^ Additional specialized dendrimer types include chiral dendrimers, peptide dendrimers, glycodendrimers, PAMAM-organosilicon (PAMAMOS) dendrimers, Fréchet-type dendrimers, hybrid dendrimers, and polyester dendrimers, each tailored for specific applications.^148,149^

**Fig. 23:**
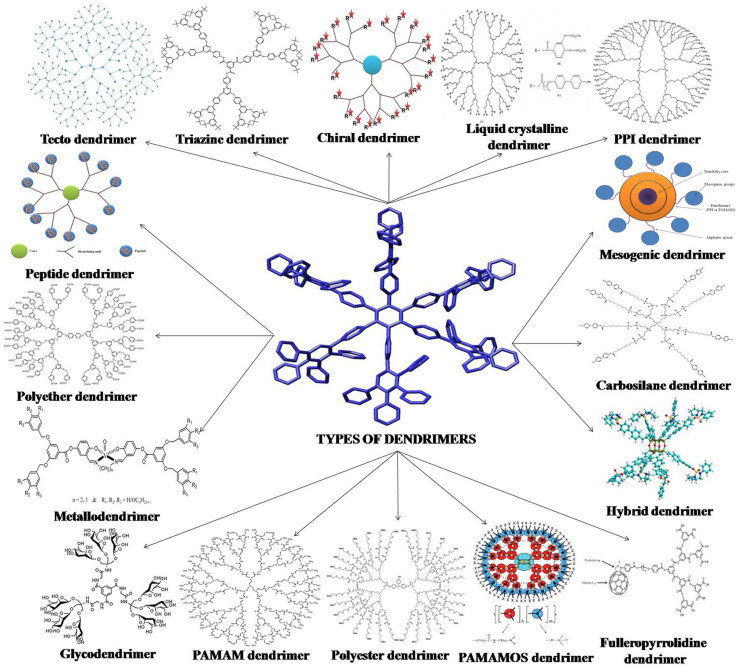
Illustration of different types of dendrimers. This figure has been reproduced from ref. 148 with permission from *Prog. Polym. Sci.*, copyright 2014.

Different types of dendrimers are illustrated in Fig. 23, highlighting their structural diversity and functional versatility in nanomedicine and drug delivery applications.

### 4.2 Preparation of dendrimers

**Fig. 24:**
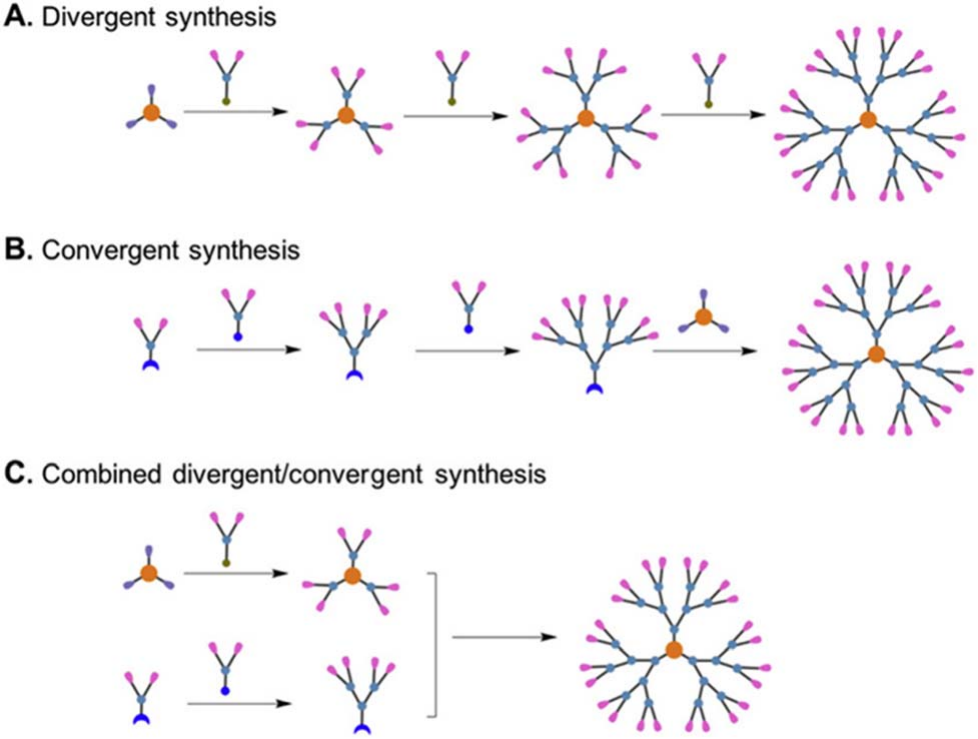
Schematic presentation of covalent dendrimer synthesis approaches: (A) divergent, (B) convergent, and (C) combined divergent-convergent. This figure has been reproduced from ref. 160 with permission from *Mater. Today Chem.*, copyright 2019.

Dendrimers are synthesized from a central core, with branches called dendrons extending through sequential chemical reactions. The precise structure of dendrimers is debated, particularly regarding whether the end groups are fully extended, maximizing surface density, or fold inward to form a densely packed interior.^151^ Their stepwise synthesis from branched monomer units allows precise control over molecular size, shape, density, polarity, flexibility, and solubility by selecting specific building units and surface functional groups.^152^ Two primary synthesis methods are employed: the divergent approach, which builds from the core outward to the periphery, and the convergent approach, which constructs dendrimers from the periphery inward (Fig. 24).^153^

#### 4.2.1 Divergent synthesis

The divergent synthesis method, pioneered by Tomalia, constructs dendrimers by growing them outward from a central core.^154^ This approach involves two key steps: activation of functional surface groups and addition of branching monomer units.^155^ Starting with a core molecule, a reagent with at least two protecting or branching sites is reacted with the core, followed by deprotection to form the first-generation dendrimer. This cycle is repeated to achieve the desired size and generational structure.^149^ Notably, PAMAM dendrimers are commonly synthesized using this method. The divergent approach allows precise tailoring of the dendrimer's surface through end-group modification, enabling customization of chemical and physical properties for applications such as targeted drug delivery and imaging.^156–158^

#### 4.2.2 Convergent synthesis

The convergent synthesis method constructs dendrimers by starting from the periphery and working inward, linking surface units to form dendrons that are subsequently attached to a central core to create a complete dendrimer.^154^ This process involves two key stages: iterative coupling of protected or deprotected branches to produce a functionalized dendron, followed by a divergent core-anchoring step to form multi-dendron dendrimers. Compared to the divergent approach, convergent synthesis offers superior control over molecular weight and precise placement of functional groups, resulting in symmetric, well-defined dendrimers with uniform distribution of functional moieties. This method minimizes side reactions, requires fewer reagents, and simplifies purification, yielding high-purity dendrimers suitable for advanced applications. However, challenges in synthesizing large quantities due to repeated reactions requiring active site protection remain a significant limitation.^149,159^

### 4.3 Dendrimers in drug delivery

Dendrimers are highly effective drug delivery vehicles due to their ability to enhance the pharmacokinetic and pharmacodynamic properties of drugs, improving bioavailability and enabling controlled, targeted release.^154^ Drugs can be encapsulated within the internal cavities of dendrimers, particularly in low-generation structures with open conformations, increasing the water solubility of hydrophobic drugs.^147,154,161^ Alternatively, the high density of surface functional groups, such as amines or carboxyls, allows covalent conjugation of drugs, with release triggered by chemical or enzymatic cleavage of hydrolytically labile bonds, offering superior control over drug release compared to simple encapsulation or electrostatic complexation.^147^ Dendrimers with hydrophobic cores and hydrophilic peripheries function as unimolecular micelles, effectively solubilizing hydrophobic drugs by entrapping them within intramolecular cavities.^162,163^

**Fig. 25:**
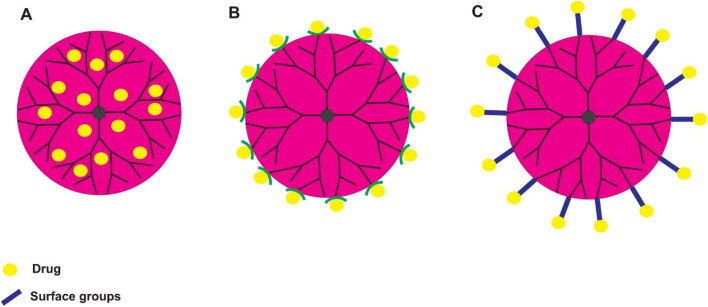
Schematic view of drug–dendrimer interactions: (A) physical encapsulation, (B) electrostatic interactions, and (C) covalent binding. This figure has been reproduced from ref. 149 with permission from *J. Inorg. Organomet. Polym. Mater.*, copyright 2021.

Dendrimers facilitate drug delivery through two primary mechanisms. The first involves *in vivo* degradation of drug–dendrimer conjugates, where covalently bound drugs are released *via* enzymatic cleavage or in response to specific environmental conditions.^154^ The second mechanism relies on changes in physical conditions, such as pH or temperature, triggering drug release from the dendrimer's core (endo-receptor) or outer shell (exo-receptor), independent of external factors.^154^ The positively charged surfaces of dendrimers enable strong interactions with negatively charged cell membranes, enhancing intracellular drug delivery. Key properties, including the ability to encapsulate or conjugate high-molecular-weight drugs, high cellular uptake, extended drug circulation time, increased drug stability, and targeted tissue delivery, make dendrimers highly effective drug carriers.^149^ Drug–dendrimer interactions, including physical encapsulation, electrostatic interactions, and covalent binding, are schematically illustrated in Fig. 25.

#### 4.3.1 Oral drug delivery

Dendrimers are promising vehicles for oral drug delivery, enhancing transepithelial permeability through both unmodified and surface-modified structures.^149^ For instance, a conjugate of the anticancer drug 7-ethyl-10-hydroxy-camptothecin (SN38) with generation 3.5 poly(amidoamine) (PAMAM) dendrimers, featuring carboxylate end groups, demonstrated improved oral bioavailability and reduced toxicity.^164^ PEGylated dendrimers have been highlighted as effective carriers when drug conjugation and release require increased surface functional groups.^165^ Jevprasesphant *et al.* showed that both unmodified and surface-modified PAMAM dendrimers efficiently crossed Caco-2 cell monolayers *via* transcellular and paracellular pathways.^166^ To address the toxicity of 5-fluorouracil (5-FU), Tripathi *et al.* modified PAMAM dendrimers with fatty acid molecules, coated them with phospholipids, and encapsulated 5-FU. *In vivo* studies in albino rats revealed significantly improved pharmacokinetic parameters and bioavailability compared to free 5-FU, demonstrating the potential of dendrimer-based formulations for effective oral drug delivery.^167^

#### 4.3.2 Transdermal drug delivery

Dendrimers enhance transdermal drug delivery by addressing the poor water solubility of hydrophobic drugs and leveraging their high water solubility and biocompatibility.^148^ These properties improve drug pharmacokinetic profiles and facilitate efficient delivery across biological barriers.^149^ Cheng *et al.* developed conjugates of ketoprofen and diflunisal with generation 5.0 PAMAM dendrimers, demonstrating 3.4- and 3.2-fold higher permeation rates, respectively, compared to drug dispersions in saline during *in vitro* studies with excised rat skin. *In vivo* anti-nociceptive studies in mice showed that the ketoprofen–dendrimer complex reduced writhing activity for 1–8 hours, compared to 4–6 hours for ketoprofen dispersion, highlighting dendrimers as an attractive approach for transdermal delivery of nonsteroidal anti-inflammatory drugs (NSAIDs).^168^ Similarly, Jain *et al.* investigated generation 4.0 (amino- and hydroxyl-terminated) and 4.5 PAMAM dendrimers for transdermal delivery of indomethacin. *In vivo* studies in Wistar rats revealed significantly higher blood concentrations of indomethacin with dendrimer-mediated delivery compared to pure drug suspension.^169^ Castro *et al.* employed PAMAM G3 dendrimers functionalized with caffeic acid (GPG3Ca) for skin wound healing. The system was tested on HaCaT keratinocyte cells (*in vitro*) and skin tissue (*in vivo*). It improved caffeic acid stability and bioavailability, showing non-toxicity and enhancing keratinocyte migration, re-epithelialization, and collagen deposition.^170^

#### 4.3.3 Ocular drug delivery

Ocular drug delivery faces challenges due to poor bioavailability caused by tear turnover and nasolacrimal drainage.^148^ Dendrimers improve drug retention and transport in ocular tissues. Vandamme and Brobeck demonstrated that PAMAM dendrimers with carboxyl or hydroxyl end groups enhanced pilocarpine retention in the eye, supporting their potential for ocular delivery.^171^ Yavuz *et al.* developed PAMAM dendrimer–dexamethasone conjugates, which exhibited improved drug transport across scleral and corneal tissues, enhancing ocular bioavailability.^172^ Alshammari *et al.* developed PAMAM G5 dendrimers for retinal delivery of the protein kinase C-β inhibitor ruboxistaurin (RBX), aimed at treating diabetic retinopathy. The formulation showed high drug-loading efficiency (up to 97.6% for a 5 : 1 RBX : PAMAM ratio), controlled *in vitro* release, and non-toxicity on MIO-M1 retinal Müller glial cells. The mechanism involves non-invasive delivery *via* dendrimer nanoparticles, enhancing RBX bioavailability and therapeutic effect while avoiding complications of intravitreal injections.^173^

#### 4.3.4 Pulmonary drug delivery

Dendrimers are promising carriers for pulmonary drug delivery, enhancing bioavailability without compromising lung function. Studies have shown that generation 2.0 and 3.0 cationic dendrimers increased the relative bioavailability of enoxaparin, a low molecular weight heparin, by approximately 40% without adversely affecting mucociliary transport or causing significant lung tissue damage.^148^ For chemotherapeutic applications, direct inhalation of uncomplexed cytotoxic drugs can lead to bolus release and lung toxicity. Kaminskas *et al.* demonstrated that DOX conjugated to PEGylated polylysine dendrimers significantly enhanced drug exposure to lung metastases, improving therapeutic outcomes compared to intravenous administration.^174^

#### 4.3.5 Gene delivery

Dendrimers, particularly amino-terminated poly(amidoamine) (PAMAM) and poly(propylene imine) (PPI) variants, serve as effective non-viral vectors for gene therapy.^154^ Compared to branched polyethylenimine (PEI), PAMAM dendrimers offer higher biocompatibility and greater nucleic acid loading capacity.^175^ Dendriplexes, complexes of dendrimers with nucleic acids such as oligonucleotides, genes, aptamers, or siRNA, exhibit high transfection efficiency.^148^ The flexible, hyperbranched architecture of dendrimers enables the formation of compact DNA complexes, facilitating cellular uptake *via* endocytosis. Once internalized, the dendrimer–DNA complex is transported to the nucleus, where DNA replication, transcription, and mRNA release occur, leading to target protein translation.^148^ Gorzkiewicz *et al.* synthesized lysine-based dendrimers (D3K2 and D3G2) and evaluated their transfection potential in HeLa (cervix adenocarcinoma) and HMEC-1 (microvascular endothelial) cell lines. The cationic D3K2 dendrimer demonstrated high transfection efficiency, enhancing intracellular accumulation of large nucleic acid molecules like plasmids with selective cytotoxicity toward cancer cells and minimal toxicity to normal cells.^176^

#### 4.3.6 Topical and antimicrobial delivery

Dendrimers are emerging as effective topical antimicrobial agents, particularly polylysine dendrimers for combating herpes simplex virus (HSV). SPL7013 gel (VivaGel®), developed by Starpharma Pty Ltd., is a polylysine dendrimer-based vaginal microbicide designed to prevent and treat HIV and HSV infections.^177^ Polylysine dendrimers modified with sulfonated naphthyl groups exhibit antiviral activity against HSV, potentially reducing transmission of HSV, HIV, and other sexually transmitted diseases.^4^ Wang *et al.* investigated the antimicrobial mechanism of PAMAM dendrimers in a guinea pig model of *Escherichia coli*-induced chorioamnionitis, attributing their efficacy to interactions between polycationic dendrimers and polyanionic lipopolysaccharides in *E. coli*.^178^ Recently, Fu *et al.* reviewed silver nanoparticle (Ag NP)-dendrimer nanocomposites as emerging antibacterial and biomedical therapeutics. Dendrimers, with their highly branched and uniform structure, stabilize Ag NPs and enhance their antibacterial activity. These nanocomposites are designed to kill drug-resistant bacteria, inhibit microbial formation, and offer potential anticancer effects, representing a promising approach for next-generation theranostic nanomedicine.^179^

#### 4.3.7 Cancer therapy

Dendrimer–polymer conjugates, incorporating PEG, polysaccharides, or polypeptides, enhance the stability and solubility of therapeutics. Polysaccharide–dendrimer conjugates, using materials like chitosan, hyaluronic acid, cyclodextrin, or dextran, improve binding properties and biocompatibility. For instance, hyaluronic acid-conjugated PAMAM dendrimers enhance tumor penetration by targeting CD44 receptors overexpressed on tumor cells and cancer stem cells.^146^ Wu *et al.* developed generation 5 (G5) PAMAM dendrimers conjugated with cetuximab and approximately 1100 boron atoms for boron neutron capture therapy, achieving 10-fold higher accumulation in brain tumor tissues compared to healthy tissues, demonstrating efficacy for intracerebral glioma treatment.^180^ Narsireddy *et al.* synthesized dual-functionality dendrimers modified with nitrilotriacetic acid (NTA) and porphine (PS) groups, incorporating a peptide specific to human epidermal growth factor receptor 2 (HER2). These PS–dendrimers enabled targeted photodynamic therapy, reducing tumor size in a HER2-positive SKOV-3 xenograft model, highlighting their potential for apoptosis induction.^181^ Dhull *et al.* developed a generation 4 hydroxy PAMAM dendrimer (PD) conjugated with prostate-specific membrane antigen (PSMA) ligand (PD-CTT1298) for targeted prostate cancer therapy. The system selectively delivers chemotherapeutics, such as cabozantinib, to PSMA-positive prostate cancer cells *via* PSMA-mediated internalization. *In vitro* and *in vivo* studies showed enhanced drug uptake, improved anti-proliferative activity, selective tumor targeting, and reduced off-target accumulation.^182^

A summary of the dendrimers discussed above for drug delivery applications is provided in Table 7.

**Table 7:** Applications of dendrimers in drug delivery

Dendrimer type/conjugate	Drug/agent	Indication	Explanation	References
Generation 3.5 poly(amido amine) dendrimers (G 3.5 PAMAM)	7-Ethyl-10-hydroxy-camptothecin (SN38)	Hepatic colorectal cancer metastases (HT-29 cells)	Conjugation of SN38 to G3.5 dendrimers increased the transepithelial transport while simultaneously reducing intestinal toxicity compared to free SN38, illustrating the potential for these conjugates in oral drug delivery	164
PAMAM dendrimers modified with fatty acid (phospholipid)	5-fluorouracil (5-FU)	Cancer cell	The dendrimer–drug formulation was found to be significantly more effective than free drugs after oral administration	167
5.0 G PAMAM dendrimer	Ketoprofen and diflunisal (nonsteroidal anti-inflammatory drugs (NSAIDs))	Pain/inflammation	Enhanced transdermal permeation (3.4× and 3.2× higher than free drug) and prolonged anti-nociceptive effect	168
4.0 G PAMAM dendrimers with amino and hydroxyl terminal	Indomethacin	Pain/inflammation	A significant increase in concentration of indomethacin in blood was observed with PAMAM dendrimer-mediated delivery of indomethacin compared to pure drug suspension	169
PAMAM G3 dendrimer gel	Caffeic acid (GPG3Ca)	HaCaT keratinocyte cells (*in vitro*) and skin tissue (*in vivo*)	It improved caffeic acid stability and bioavailability, showing non-toxicity and enhancing keratinocyte migration, re-epithelialization, and collagen deposition	170
PAMAM dendrimer with carboxyl/hydroxyl ends	Pilocarpine nitrate and tropicamide	Ocular disorders	Increased retention in eyes, supporting ocular delivery	171
PAMAM dendrimer	Dexamethasone	Diabetic retinopathy	Increased drug transport across sclera and cornea	172
(PAMAM) dendrimer generation 5	Ruboxistaurin (RBX)	Diabetic retinopathy	The formulation showed high drug-loading efficiency, controlled *in vitro* release, and non-toxicity on MIO-M1 retinal Müller glial cells	173
PEGylated polylysine dendrimers	DOX	Lung-resident cancers	Increased exposure to lung metastases	174
Lysine-based dendritic macromolecules (D3K2 and D3G2)	Plasmids (nucleic acid)	Two human cell line models: cervix adenocarcinoma (HeLa) and microvascular endothelial (HMEC-1)	The dendrimers exhibited specific cytotoxicity towards cancer cell line without showing significant toxic effects on normal cells	176
PAMAM dendrimers	Monoclonal antibody (MoAb), cetuximab, as a boron delivery agent for neutron capture therapy (NCT)	Wild-type and mutant VIII isoform of the epidermal growth factor receptor (EGFR) in intracerebral glioma	The *in vivo* results revealed that the accumulation of conjugates was 10 times higher in brain tumor tissues in comparison to healthy brain tissues	180
PAMAM dendrimers conjugated with nitrilotriacetic acid (NTA) group and a peptide specific to human epidermal growth factor receptor 2 (HER2)	Efficient near infrared-sensitive photosensitizers (5,10,15,20-tetrakis(4-hydroxyphenyl)-21*H*,23*H*-porphine [PS])	The human SK-OV-3 cell line overexpressing HER2 receptors (HER2^+^ cells) and MCF7 cell line (HER2^−^ cells)	Significant improvement in photodynamic therapy (PDT) efficacy compared to free PS	181
Generation 4 hydroxy PAMAM dendrimer (PD) conjugated with prostate specific membrane antigen (PSMA) ligand (PD-CTT1298)	Cabozantinib	PSMA-positive prostate cancer cells	*In vitro* and *in vivo* studies showed enhanced drug uptake, improved anti-proliferative activity, selective tumor targeting, and reduced off-target accumulation	182

### 4.4 Dendrimers in medical imaging

Dendrimers enhance medical imaging by encapsulating or conjugating bioimaging agents, either within their internal cavities *via* physical entrapment or non-covalent interactions (electrostatic, hydrophobic, or hydrogen bonding) or through covalent linkages (*e.g.*, amide or ester bonds) to their surface.^150^ Their versatility makes them ideal for applications in CT, MRI, radiotherapy, and as molecular probes.^148^

#### 4.4.1 Dendrimers in CT and MRI imaging

Dendrimer-based contrast agents offer advantages such as high iodine content, prolonged intravascular enhancement, high X-ray attenuation, low osmolality, and excellent chemical stability and hydrophilicity.^148^ For CT imaging, a generation 5 (G5) PAMAM dendrimer conjugated with FA and fluorescein thiocyanate, with acetylated amine terminals, was used to synthesize AuNPs within its cavities. This nanosystem demonstrated specific accumulation in folate-overexpressing cervical cancer cells, enabling effective micro-CT imaging *in vivo* with minimal cytotoxicity.^183^ Fu *et al.* developed CT-compatible paired dendrimers with a PEG core (G3–G5, 16–64 amino groups) conjugated with triiodophthalamide moieties. In rat models, these dendrimers exhibited high water solubility, low osmolality, and a 35 minute half-life for G4 molecules, achieving successful intravascular imaging.^184^ In MRI, dendrimers enhance tumor-targeting and relaxivity when conjugated with gadolinium.^149^ Yordanov *et al.* used gadolinium-conjugated dendrimers to achieve vessel-specific enhancement in 2D MR imaging of SCCVII tumors in murine models without diffuse enhancement.^185^ Zhang *et al.* synthesized biocompatible oligo(ethylene glycol) (OEG)-based radical dendrimers (G1 and G2) with high molecular relaxivity and full water solubility at physiological pH. The G1-OEG-PROXYL dendrimer exhibited a relaxivity (3.4 mM^−1^ s^−1^) comparable to clinical Gd-DTPA (3.2 mM^−1^ s^−1^ at 7T), avoiding toxicity risks associated with gadolinium accumulation.^186^

#### 4.4.2 Multimodality imaging agents

**Fig. 26:**
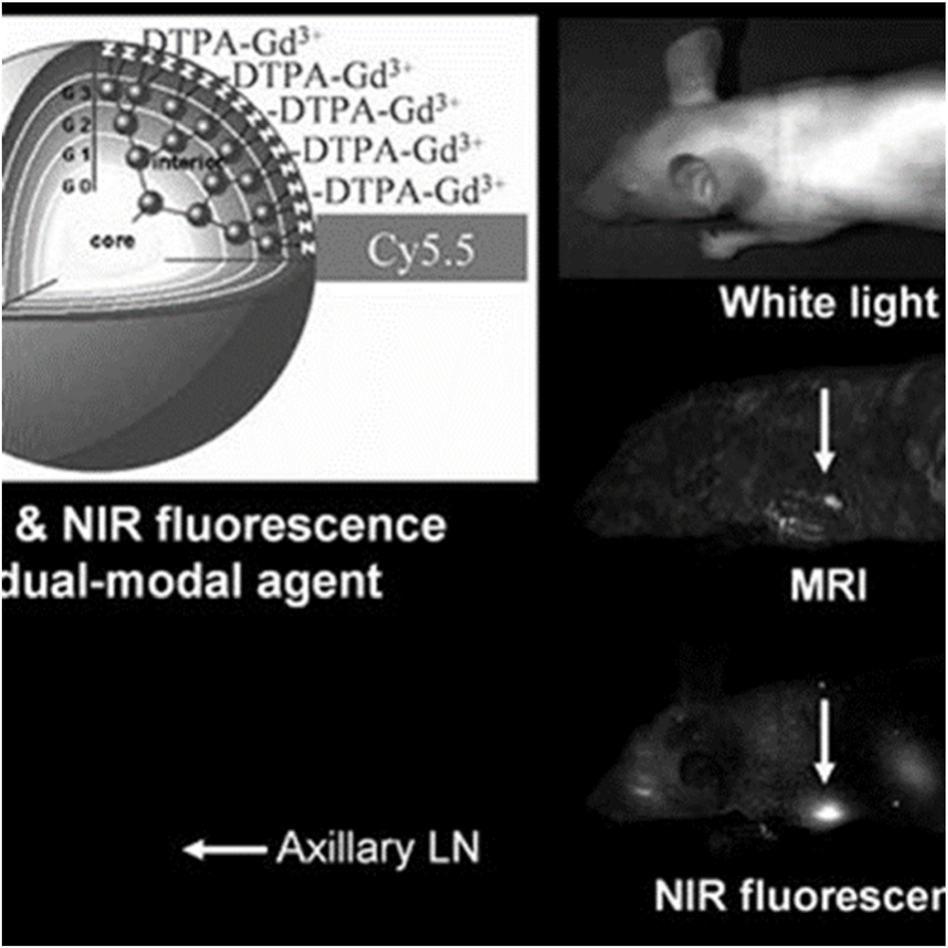
MRI and near-infrared fluorescence imaging obtained with a dendrimer-based dual-modal contrast agent. This figure has been reproduced from ref. 188 with permission from *J. Magn. Reson. Imaging*, copyright 2007.

Dendrimers are ideal platforms for multimodality imaging due to their numerous attachment sites, enabling the incorporation of multiple imaging beacons on a single molecule. A novel dual-modality magnetic resonance (MR) and fluorescence imaging agent was developed by conjugating Gd(iii)-1B4M-DTPA to a generation 2 (G2) PAMAM dendrimer, followed by attaching a maleimide-functionalized biotin after reducing a core disulfide bond. This biotinylated dendrimer was optimized for optical imaging by binding to fluorescently labeled avidin, forming a supramolecular avidin-biotin-dendrimer-Gd(iii) complex. This system effectively targeted disseminated peritoneal ovarian tumors, delivering chelated Gd(iii) and fluorophores like Rhodamine Green for dual MR and fluorescence imaging.^187^ Similarly, a generation 6 (G6) PAMAM dendrimer labeled with Cy5.5 NIR fluorophore and Gd(iii) was developed as a macromolecular MR/NIR optical contrast agent, as shown in Fig. 26. When injected into the mammary glands of healthy mice, this agent enabled precise identification of SLNs *via* both MR and optical imaging, with MR providing superior resolution near the injection site.^188^

**Fig. 27:**
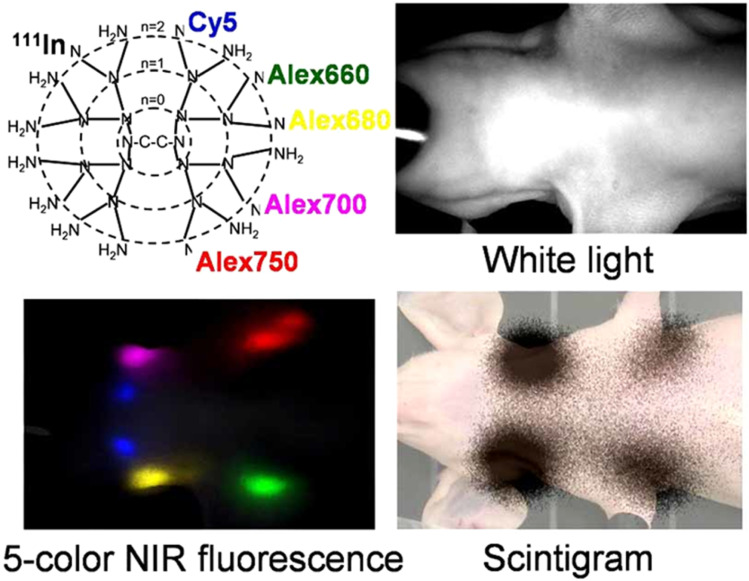
Scintigraphy and five-color near-infrared fluorescence imaging obtained with dendrimer-based dual-modality multicolor contrast agents. This figure has been reproduced from ref. 190 with permission from *ACS Nano*, copyright 2007.

Wu *et al.* developed a water-soluble, bimodal G2 polyamide dendrimer integrating TEMPO organic radicals for magnetic resonance imaging (MRI) and a 1,8-naphthalimide (Naft) fluorophore for fluorescence imaging (FI). This dendrimer successfully stains mesenchymal stem cells (MSCs) without nuclear toxicity and demonstrates MRI contrast (*r*_1_ = 1.3 mM^−1^ s^−1^). The mechanism relies on radical–fluorophore interactions with partial fluorescence quenching *via* photoinduced electron transfer (PET), while maintaining effective dual imaging capabilities, highlighting its potential as a metal-free bimodal imaging probe for biomedical diagnostics.^189^ Dendrimers enable advanced multimodality imaging by integrating multiple imaging beacons into a single carrier. A generation 6 (G6) PAMAM dendrimer labeled with a radioisotope and five-color NIR fluorophores was developed for lymphatic imaging. This radionuclide-optical combination allowed a 100-fold reduction in injected dose compared to MRI-optical agents, achieving comparable sensitivity to clinical sentinel node imaging agents (Fig. 27).^190^

**Fig. 28:**
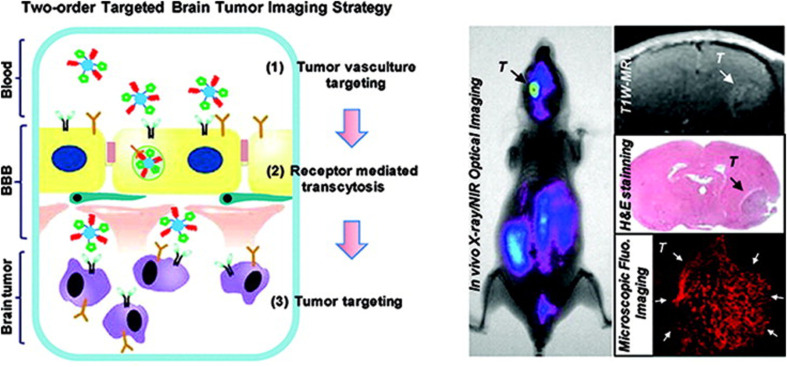
Design of the two-order targeted nanoprobe (MR/optical imaging) for brain tumor imaging. This figure has been reproduced from ref. 191 with permission from *ACS Nano*, copyright 2012.

To overcome the BBB for brain tumor imaging, a dual-targeted nanoprobe was engineered using a G5 PAMAM dendrimer conjugated with MR/optical imaging reporters and tumor vasculature-targeted cyclic (RGDyK) and angiopep-2 peptides, as shown in Fig. 28. This nanoprobe targets αVβ3 integrin on tumor vasculature and LRP receptors on endothelial cells, facilitating BBB crossing *via* receptor-mediated endocytosis. *In vivo* imaging in mice demonstrated efficient BBB traversal and precise delineation of orthotopic U87MG glioblastoma boundaries with a high target-to-background ratio, offering potential for non-invasive brain tumor visualization and real-time surgical guidance.^191^

For atherosclerosis (AS) imaging, a chronic inflammatory vascular disease linked to myocardial infarction and cerebrovascular events, Seo *et al.* developed a (ARAL)_4_-dendrimer-^64^Cu nanoprobe with a lysine core, targeting p32 protein overexpressed in macrophage-rich AS lesions. In ApoE atherosclerotic mice, this nanoprobe exhibited significantly higher uptake in the aortic root and descending aorta compared to a control (ARAL)_4_-dendrimer-^64^Cu, as confirmed by PET-CT and *ex vivo* imaging at 2–3 hours post-injection. Subcutaneous injections further showed preferential accumulation in plaque-rich regions over 24 hours, highlighting its potential as a PET imaging nanocarrier for AS.^192^

Table 8 shows a summary of the dendrimers discussed above for medical imaging applications.

**Table 8:** Applications of dendrimers in medical imaging

Dendrimer type/conjugate	Detection	Imaging technique	References
G5 PAMAM conjugated with FA and fluorescein thiocyanate and acetylated with AuNPs	HeLa cervical cancer	CT	183
PEG-core dendritic polylysine (G3, G4 and G5) conjugated with triiodophthalamide and iodinated contrast agents	Vascular imaging	CT	184
PAMAM dendrimer-based biometric nanoprobes G4-1B4M-Gd, G6-1B4M-Gd and G8-1B4M-Gd	SCCVII tumor	MRI	185
Oligo(ethylene glycol) dendrimers functionalized with 5 and 20 PROXYL organic radicals and gadolinium (Gd)-based contrast agents (CA)	General tumor imaging	MRI	186
Avidin-biotin-PAMAM dendrimer-Gd(iii) complex	Ovarian tumors	MRI/fluorescence	187
G6 PAMAM dendrimer, labeled with Cy5.5 NIR fluorophore coupled with a Gd	SLNs	MRI/NIR fluorescence	188
Aminoterminated polyamide dendrimer containing a 1,8-naphthalimide (Naft) fluorescent group and TEMPO organic radicals as terminal groups	C57BL/6 mouse bone marrow mesenchymal stem cells (MSCs)	MRI/fluorescence	189
G6 PAMAM dendrimer with radioisotope and NIR fluorophore	Lymphatic imaging	Radionuclide/fluorescence imaging	190
PAMAM dendrimer (G5) conjugated with MR/optical imaging reporters and tumor vasculature-targeted cyclic (RGDyK) and angiopep-2 peptides	αVβ3 integrin and LRP receptors in brain tumor cells, particularly U87MG glioblastoma	MRI/optical imaging	191
Dendrimer with multiple LyP-1 ligands, using lysine as the core, termed (LyP-1)_4_-dendrimer-^64^Cu	p32 protein as an overexpressed biomarker in atherosclerosis (AS)	PET-CT	192

### 4.5 Dendrimers in biosensors

**Fig. 29:**
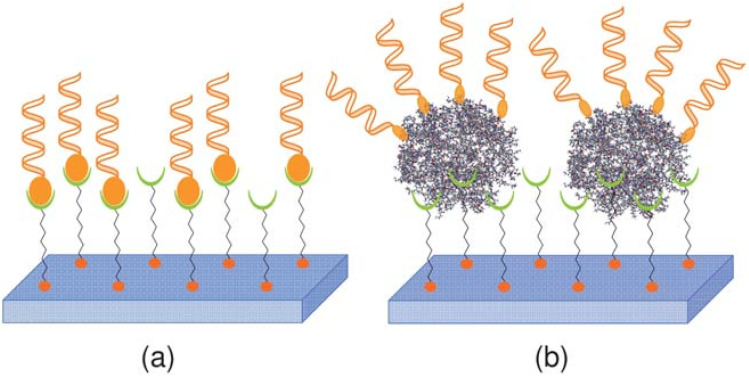
Cartoon illustrating the immobilization efficiency of ssDNA on (a) linear linker and (b) dendrimer-derived substrate. This figure has been reproduced from ref. 194 with permission from *J. Mater. Chem.*, copyright 2011.

Dendrimers are versatile platforms for biosensing, integrated with transducers such as electrochemical, optical, or quartz crystal microbalance (QCM) systems to detect analytes such as DNA, drugs, proteins, and microorganisms with high sensitivity and specificity.^193^ Nucleic acid dendrimers enhance the performance of DNA biosensors by providing a large number of single-stranded DNA (ssDNA) arms, significantly increasing hybridization capacity (Fig. 29). These dendrimer-derived matrices exhibit at least eightfold improved sensitivity and detection limits compared to conventional oligonucleotide-based biosensors, with negligible responses to noncomplementary oligomers.^194^ Surface modification techniques, including Langmuir–Blodgett, noncovalent interactions, covalent attachment, and spin casting, have been employed to graft dendrimers onto sensor substrates like gold or silica (*e.g.*, glass slides, silicon wafers, and silica fibers), optimizing their biosensing capabilities.^194^

#### 4.5.1 Electrochemical biosensors

**Fig. 30:**
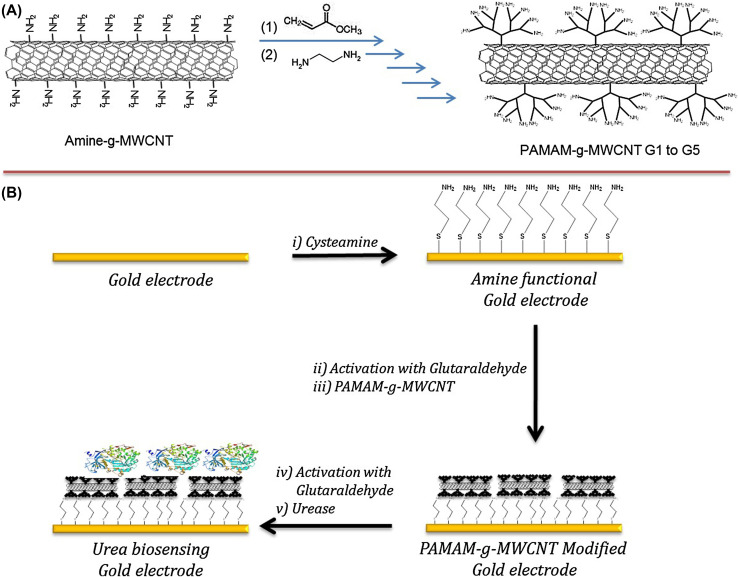
(A) Experimental route for synthesis and modification of MWCNT-PAMAM. (B) Schematic illustration of the preparation of the urea biosensing electrode. This figure has been reproduced from ref. 195 with permission from *Sens. Actuators, B*, copyright 2018.

Dervisevic *et al.* developed an amperometric urea biosensor by immobilizing urease enzyme onto PAMAM-grafted multi-walled carbon nanotube (MWCNT-PAMAM) dendrimers (G1 to G5) on a gold electrode (Fig. 30). The G5 MWCNT-PAMAM-urease electrode exhibited exceptional performance, with a rapid 3 second response time, a wide linear detection range of 1–20 mM, a detection limit of 0.4 mM, and a sensitivity of 6.6 nA mM^−1^. The biosensor showed negligible interference from potential contaminants, highlighting its specificity and potential for precise urea detection.^195^

Dendrimer-based electrochemical biosensors leverage the unique properties of dendrimers to enhance sensitivity and specificity in analyte detection. A glucose biosensor was developed by conjugating glucose oxidase (GO_*x*_) with polyglycerol (PGLD) and chitosan dendrimers, entrapped within polyaniline nanotubes (PANINTs) during aniline electrochemical polymerization. The PGLD-GO_*x*_/PANINTs and CHD-GO_*x*_/PANINTs biosensors exhibited robust amperometric responses to glucose at +100 mV, effectively detecting glucose within the human blood concentration range (0.02–10 mM). The PGLD-GO_*x*_/PANINTs biosensor showed higher sensitivity (10.41 nA mM^−1^) compared to CHD-GO_*x*_/PANINTs (7.04 nA mM^−1^), attributed to the optimized GO_*x*_ layer organization on PGLD, while both maintained strong enzyme affinity (*K*^app^_M_) post-immobilization, enabling nanoscale fabrication for biocompatible implants and high-throughput testing.^196^ Armada *et al.* developed amperometric glucose and hydrogen peroxide biosensors using polymethylferrocenyl dendrimers with poly(propylene imine) cores functionalized with octamethylferrocenyl units, deposited on platinum electrodes, demonstrating effective redox-mediated detection.^197^ Shim *et al.* engineered electrocatalytic bioassays for ultrasensitive protein and DNA detection using generation 3 (G3) PAMAM dendrimers with a poly-5,20 : 50 200-terthiophene-30-carboxylic acid (pTTCA) matrix and AuNPs/CdS NPs. This platform, utilizing H_2_O_2_ reduction catalyzed by hydrazine labels, achieved detection limits of 450 aM for DNA and 4 fg mL^−1^ for proteins, with over 70-fold enhanced sensitivity due to high AuNP loading. Adapted for competitive immunoassays, it detected Annexin II (51 pg mL^−1^) and chloramphenicol (45 pg mL^−1^). Additionally, hydroxyl-terminated PAMAM dendrimers with rhodium nanoparticles (RhNPs) enabled sensitive dopamine detection in urine, underscoring the versatility of dendrimer-based biosensors.^198–200^

#### 4.5.2 Optical biosensors

**Fig. 31:**
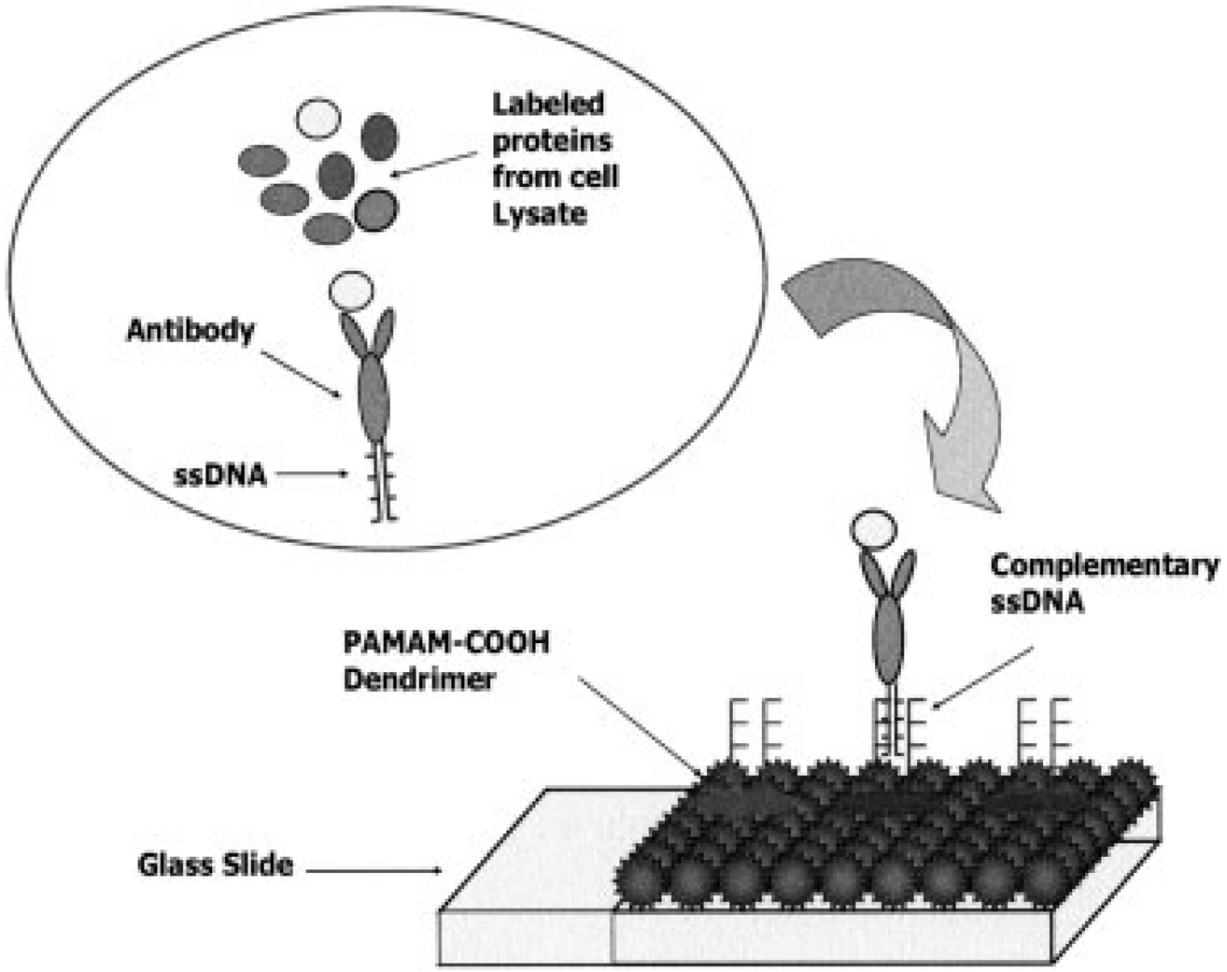
Cartoon representation of the SAPA platform, illustrating ssDNA-tagged antibody capture of labeled antigens and spatial addressability on PAMAM carboxyl-derivatized glass surfaces. This figure has been reproduced from ref. 201 with permission from *Electrophoresis*, copyright 2007.

Dendrimers enhance optical biosensing by enabling ultrasensitive fluorescence detection with limits as low as 1 pM, attributed to high loading efficiency, uniform bioreceptor distribution, and reduced fluorescence quenching due to optimal spacing on dendrimeric matrices.^194^ These properties make dendrimers ideal for surface functionalization in protein and DNA/RNA microarrays, immobilized *via* electron beam lithography or microcontact printing with a dendri-stamp using click chemistry. Such microarrays exhibit uniform spot morphology and consistent receptor–ligand interactions, facilitating fluorescence-based detection of proteins and nucleic acids.^194^ Ng *et al.* developed a spatially addressable protein array (SAPA) using carboxylated PAMAM dendrimer-modified surfaces for ssDNA-directed assembly of antibody–antigen complexes, as shown in Fig. 31. This platform enabled sensitive antigen detection in dilute biological samples, leveraging specific ssDNA hybridization for enhanced capture efficiency.^201^

In surface plasmon resonance (SPR) biosensors, a typical matrix comprises an alkane thiol self-assembled monolayer (SAM) on a gold surface, a PAMAM dendrimer intermediate layer, and a biomolecular recognition layer. This configuration overcomes mass transport limitations, achieving a detection limit of 3.9 nM for target DNA with an ultrathin underlying amino-undecanethiol (AUT) SAM/PAMAM/pDNA film, while also improving stability and bioreceptor layer regeneration.^202^ Jung *et al.* enhanced surface plasmon resonance (SPR) detection of foot-and-mouth disease (FMD) peptides using PAMAM succinamic acid dendrimers conjugated with aptamers. The dendrimer-conjugated aptamer layer on the Au SPR surface increased detection sensitivity by 20-fold, lowering the limit of detection (LOD) to 0.01 nM compared to 0.1 nM without dendrimers.^203^

#### 4.5.3 QCM biosensors

Quartz crystal microbalance (QCM) biosensors detect mass changes on a quartz crystal surface by measuring shifts in oscillation frequency, with antigen–antibody interactions increasing mass and decreasing frequency.^193^ Svobodová *et al.* developed a QCM immunosensor for human IgG detection by immobilizing *anti*-IgG antibodies on generation 4 (G4) PAMAM dendrimers combined with 1-hexadecanethiol (HDT) layers or protein A. The G4 PAMAM-HDT self-assembled monolayers enabled sensitive detection of human IgG with a low detection limit, demonstrating their potential for precise immunosensing.^204^ Nisiewicz *et al.* developed an ultrasensitive immunosensor based on G2 PAMAM dendrimers for the detection of matrix metalloproteinase-9 (MMP-9) in plasma. Two platforms were compared: PAMAM-NH_2_ and PAMAM-COOH. The PAMAM-NH_2_ immunosensor achieved the best performance with a limit of detection (LOD) of 2.0 pg mL^−1^ and a wide analytical range (1 × 10^−4^ to 5 μg mL^−1^). Detection relied on antigen–antibody recognition monitored *via* QCM-D and EIS, and the device showed high recovery (96–120%) in spiked human and rat plasma.^205^

A summary of the dendrimers discussed above for biosensors applications is provided in Table 9.

**Table 9:** Applications of dendrimers in biosensors

Dendrimer type/conjugate	Detection	Explanation	References
Urease enzyme onto poly(amidoamine) grafted multi-walled carbon nanotube (MWCNT-PAMAM) dendrimers (from G1 to G5) on Au electrode (electrochemical sensor)	Urea	Amperometric biosensor with 0.4 mM detection limit, 6.6 nA mM^−1^ sensitivity	195
Polyglycerol (PGLD) and chitosan (CHD) dendrimers bioconjugated with glucose oxidase (GO_*x*_) entrapped within polyaniline nanotubes (PANINTs)	Glucose	Amperometric biosensor with 10.41 nA mM^−1^ (PGLD) and 7.04 nA mM^−1^ (CHD) sensitivity	196
Polymethylferrocenyl dendrimers deposited onto a platinum electrode and immobilization of glucose oxidase onto these modified electrodes (electrochemical sensor)	Hydrogen peroxide, glucose	These redox dendrimers consist of flexible poly(propileneimine) dendrimer cores functionalized with octamethylferrocenyl units	197
H_2_O_2_ reduction catalyzed by hydrazine labels on a poly-5,20 : 50 200-terthiophene-30-carboxylic acid pTTCA polymer matrix with 3 G PAMAM dendrimers and AuNPs/CdS NPs	DNA, proteins (Annexin II, chloramphenicol)	Ultrasensitive detection (450 aM for DNA, 4 fg mL^−1^ for proteins)	198–200
Carboxylated PAMAM with ssDNA-tagged antibody (optical biosensor for protein array *via* ssDNA hybridization)	Antigens	This platform allowed antigen detection in cell lysate with high sensitivity (1 pM)	201
PAMAM G4 succinamic-acid dendrimers conjugated with double-stranded(ds)-aptamers, formed on the Au surface *via* a thiol bond between the aptamers and Au (SPR sensor)	Foot-and-mouth disease (FMD) detection	The dendrimer-conjugated aptamer layer on the Au SPR surface increased detection sensitivity by 20-fold, lowering the limit of detection (LOD) to 0.01 nM compared to 0.1 nM without dendrimers	203
G4 PAMAM dendrimers with 1-hexadecanethiol (G4-HDT) layers and immobilized with *anti*-IgG antibodies on a gold surface (QCM immunosensor)	Detection of human IgG	With 7 nM detection limit	204
G2 PAMAM dendrimers	Detection of matrix metalloproteinase-9 (MMP-9)	The PAMAM-NH_2_ immunosensor achieved the best performance with a limit of detection (LOD) of 2.0 pg mL and a wide analytical range (1 × 10^−4^ to 5 μg mL^−1^	205

## 5 Conclusion

Polymeric nanocarriers—nanomicelles, nanogels, and dendrimers—are at the forefront of nanomedicine, transforming the way we deliver treatments, image diseases, and detect biological signals. Their astounding capabilities have been highlighted in this review: nanomicelles, formed by self-assembly of amphiphilic copolymers, can dissolve hydrophobic drugs such as doxorubicin and release them selectively according to signals such as pH or light, leveraging the tumor's leaky vasculature for targeted drug delivery *via* the enhanced permeability and retention (EPR) effect. Nanogels, whose networks expand with water, protect sensitive cargos such as insulin or nucleic acids and move past barriers such as the blood–brain barrier (BBB) to bring them to critical sites. Dendrimers, whose highly branched structures enhance imaging resolution and enable ultrasensitive detection of analytes such as glucose or DNA, have yet to overcome all challenges. Scaling up to volume production, preserving long-term biocompatibility, and meeting regulatory specifications continue to be the greatest challenges. The importance of this review is that it provides complete coverage, interconnecting the three main biomedical applications of polymeric nanomaterials in a single evaluation and facilitating direct comparisons of their distinct advantages and drawbacks. This holistic method not only facilitates interdisciplinary knowledge transfer but also helps in developing multifunctional dual/triple-functional nanoplatforms, thus directing further developments toward individualized therapy. Addressing this critical need for integration, the article provides a foundational reference for both academic research and clinical translation. Future efforts should focus on streamlining synthesis, conducting extensive safety testing, and advancing clinical trials to translate these advances to the clinic. By resolving these issues, polymeric nanocarriers have the potential to transform healthcare, with personalized therapies and diagnostics that precisely address individual patient needs.

## Author contributions

Mehdi Rajabi and Hossein Feyzbakhsh contributed equally to this work. Both authors jointly conceived the idea and defined the scope of the review. Mehdi Rajabi and Hossein Feyzbakhsh collaboratively performed the literature survey, data collection, and critical evaluation of recent advances in polymeric nanocarriers, including nanomicelles, nanogels, and dendrimers. Writing of the manuscript was carried out in close collaboration, with both authors drafting, revising, and editing all sections. Both authors approved the final version of the manuscript and agree to be accountable for all aspects of the work.

## Conflicts of interest

The authors declare that they have no competing interests.

## Data Availability

No primary research results, software, or code have been included, and no new data were generated or analyzed as part of this review.
